# Nuclear ubiquitination by FBXL5 modulates Snail1 DNA binding and stability

**DOI:** 10.1093/nar/gkt935

**Published:** 2013-10-23

**Authors:** Rosa Viñas-Castells, Álex Frías, Estefanía Robles-Lanuza, Kun Zhang, Gregory D. Longmore, Antonio García de Herreros, Víctor M. Díaz

**Affiliations:** ^1^Programa de Recerca en Càncer, Institut Hospital del Mar d’Investigacions Mèdiques (IMIM), Parc de Recerca Biomèdica de Barcelona, Doctor Aiguader, 88, E-08003, Barcelona, Spain, ^2^Departament de Ciències Experimentals i de la Salut, Universitat Pompeu Fabra, Barcelona, Spain, ^3^BRIGHT Institute, Washington University, St. Louis, MO 63110 USA, ^4^Departments of Cell Biology and Physiology, Washington University, St. Louis, MO 63110 USA and ^5^Department of Medicine, Washington University, St. Louis, MO 63110 USA

## Abstract

The zinc finger transcription factor Snail1 regulates epithelial to mesenchymal transition, repressing epithelial markers and activating mesenchymal genes. Snail1 is an extremely labile protein degraded by the cytoplasmic ubiquitin-ligases β-TrCP1/FBXW1 and Ppa/FBXL14. Using a short hairpin RNA screening, we have identified FBXL5 as a novel Snail1 ubiquitin ligase. FBXL5 is located in the nucleus where it interacts with Snail1 promoting its polyubiquitination and affecting Snail1 protein stability and function by impairing DNA binding. Snail1 downregulation by FBXL5 is prevented by Lats2, a protein kinase that phosphorylates Snail1 precluding its nuclear export but not its polyubiquitination. Actually, although polyubiquitination by FBXL5 takes place in the nucleus, Snail1 is degraded in the cytosol. Finally, FBXL5 is highly sensitive to stress conditions and is downregulated by iron depletion and γ-irradiation, explaining Snail1 stabilization in these conditions. These results characterize a novel nuclear ubiquitin ligase controlling Snail1 protein stability and provide the molecular basis for understanding how radiotherapy upregulates the epithelial to mesenchymal transition-inducer Snail1.

## INTRODUCTION

The Snail family of transcription factors controls epithelial to mesenchymal transition (EMT), a process that results in the acquisition of mesenchymal characteristics by normal epithelial cells (1,2). Expression of Snail1 causes E-cadherin inhibition and EMT, and provides cells with higher motility, increased invasion and resistance to cell death (3). Snail1 is an unstable protein and extremely sensitive to proteasome inhibitors (4). At least two RING finger ubiquitin ligases of the F-box subfamily containing the multimeric complex Skp1-Cullin-Rbx1-F-box (SCF) have been shown to target Snail1 for proteasomal degradation. SCF^β-TrCP1/FBXW1^ polyubiquitinates Snail1 after its phosphorylation by glycogen synthase kinase-3β (GSK-3β) (5). Snail1 is also targeted by the SCF^Ppa/FBXL14^; this ubiquitin ligase acts as a master regulator of the EMT process, as it modulates not only Snail1 but also Snail2, Twist1 and Zeb2 (6). Contrarily to β-TrCP1, FBXL14 does not require previous Snail1 phosphorylation by GSK-3β (4,7). Both ligases are present and act exclusively in the cytosol (4,5). Mdm2, a monomeric ring finger E3, can also degrade the Snail1 family member Snail2 (8,9).

Snail1 protein half-life and action of ubiquitin ligases is finely controlled by post-translational modifications. Besides CK1ε and CK2 (10,11), required for subsequent phosphorylation of Snail1 by GSK-3β and degradation, Snail1 is stabilized by other protein kinases such as Lats2 (12), Pak1 (13) or ATM (14); in addition, Snail1 half-life is increased by *O*-glycosylation (15) and ADP-ribosylation (16). Canonical Wnt ligands, transforming growth factor-beta or cytokines activating the NF-κB pathway also increase Snail1 protein stability (5,17–19). In addition, hypoxia stabilizes Snail1 by promoting FBXL14 downregulation (4).

In this study, we have identified FBXL5 as a new SCF ubiquitin ligase that degrades Snail1. FBXL5 is an iron-regulated ubiquitin ligase that targets the iron regulatory protein 2 (IRP2) for proteasome degradation (20,21). We have found that FBXL5 is localized in the nucleus, binds Snail1 in this compartment and ubiquitinates it, preventing its interaction with DNA and promoting its degradation.

## MATERIALS AND METHODS

### Cell culture, reagents and antibodies

Cell lines used in this study have been previously described (22,23). When indicated, cells were treated with 100 µM ferric ammonium citrate (FAC), 100 µM deferroxamine mesylate (DFX), 10 or 25 µM MG132, 20 µg/ml cycloheximide (CHX), 10 μM doxorubicin, 2.5 µg/ml actinomycin D (Act D; all from Sigma), 5 ng/ml leptomycin B (LMB; LCLabs) or 10 μM etoposide (Calbiochem). The following antibodies were used: rabbit polyclonal (pAb) Lamin B1 (sc-20682), goat pAb FBXL5 (sc-54364), rabbit pAb FBLX5 (sc-134984), rabbit pAb mSin3A (sc-994) and goat pAb Zeb1 (sc-10572), all from Santa Cruz; goat pAb GST (27-4577-01, from GE Healthcare); rabbit pAb GFP (ab6556), rabbit pAb histone H3 (ab1791), mouse mAb gamma H2AX (phospho S139) (ab22551) and rabbit mAb Skp1 (ab10546), all from Abcam; rabbit pAb HA tag (H6908) and mouse mAb tubulin (T9026), both from Sigma; rat mAb HA tag (65850900, Roche); mouse mAb V5 tag (46-0705, Invitrogen); mouse mAb FK2 (04-263, Millipore); goat pAb Pyruvate Kinase (Chemicon AB1235); mouse mAb hybridomas Snail1 (24) and Myc (clone 9E10, a gift from Dr. Gabriel Gil); and rabbit pAb FBXW5 (provided by Dr Frauke Melchior, Heidelberg, Germany) (25).

### Construction of expression vectors

The pcDNA3-Snail1-HA and GFP-Snail1 constructs (full-length, GFP-Snail1; N-terminal, GFP-Snail1NT; C-terminal, GFP-Snail1CT and C-terminal deleted in the last zinc finger, GFP-Snail1CT ΔZnF4) have been previously described (26). The pcDNA3-6xMyc-FBXL5 and 6xMyc-ΔF-FBXL5 were provided by Dr J. Wohlschlegel (20). The pcDNA3.1-V5-FBXL5 wt and ΔNt, and pCI-Flag-ΔHr-FBXL5 plasmids were provided by Dr R. Bruick and have been previously described (21). The pBabe-6xMyc-FBXL5 was obtained by subcloning the insert from the pcDNA3-6xMyc-FBXL5 vector. pcDNA-Flag-Lats2 has also been described (12). GFP-Snail-CT domain deletion constructs were prepared from peGFP-C1 Snail1-HA replacing a BglII/EcoRV insert that leaves the HA tag in frame. To delete ZnF1 and ZnF1-2, the following primers with the BglII (forward primer) or EcoRV (reverse primer) restriction sites were used: FWSna (ΔZnF1): 5′-GCAATAGATCTGCACGACCTGTGGAAAGG-3′; FWSna (ΔZnF1-2): 5′-GCAATAGATCTCCTGCTCCCACTGCAAC-3′; RVSna: 5′-CAAGATATCCGCGAGGGCCTC-3′. To delete ZnF2 to ZnF4 and ZnF3 to ZnF4, the following primers were used: FWSna: 5’-GCAATAGATCTTCAACTGCAAATATTGTAAC-3′; RVSna (ΔZnF2-4): 5′-CAAGATATCCGAATGGCTTCTCACCAG-3′; RVSna (ΔZnF3–4): 5′-CAAGATA TCCGACACAAGGCAGCGTG-3′. A BamHI/XhoI FBXL5 insert was subcloned from pFastBac1-3xFlag-FBXL5 into pGEX-6P-1 to produce recombinant GST-FBXL5 protein. The expression plasmids for Twist1 cDNA containing the V5 epitope (27) and for Zeb1 cDNA (28) have been previously described. pEBG-2T-GST-Snail1-HA was obtained after subcloning a BamHI/NotI insert from pcDNA3-Snail1-HA expression vector. pcDNA3-Snail2-HA was cloned by cDNA amplification using the forward primer 5′-CCGGATCCACCATGCCGCGCTCCTTCC-3′ and reverse primer 5′-CAAGATATCCGTGTGCCACACAGC-3′ with BamHI and EcoRV restriction sites, respectively, and cloned in the pcDNA3-HA vector. GFP-FBXL5 construct was obtained by polymerase chain reaction (PCR) amplification of the sequence from a pcDNA3-6xMyc-FBXL5 construct using the following primers: FW (XhoI restriction site) 5′-ATATCTCGAGATGCGCAAGGGGG-3′, RV (BamHI restriction site) 5′-TATGGATCCTTCGCCAGAGCGGCAG-3′ and cloned into peGFP-C1. Baculovirus expression vectors were obtained after subcloning cDNAs into pFastBac1 (Snail1-HA and FBXL14-HA as BamHI/NotI inserts from pcDNA3 expression vectors and GST-Snail1-HA as an EcoRI/NotI insert from pEBG-2T expression vector) or into pFastBacHT B with the 6×His tag (6×His-TEV-Snail1-HA as a BamHI/XhoI insert from pcDNA3 expression vector). pFastBac1 3×Flag-FBXL5 was provided by Dr. Bruick, pFastBac1-HA-Cullin1, pFastBac1-6xHis-T7-Skp1 and pFastBac1-Roc1/Rbx1 were obtained after subcloning the BamHI/NotI (Cullin1 and Skp1) or BamHI/XhoI (Roc1) inserts from pBacPAK8 vectors (gift from Dr Matsumoto). All constructs were verified by sequencing in both directions.

### RNA interference screening

Short hairpin RNAs (shRNAs) against all the described F-box ubiquitin ligases were purchased from Sigma (MISSION® shRNA plasmids) and used to produce lentiviral particles (4). Transduced cell lines were selected using puromycin (1 µg/ml for MCF7 and 4 µg/ml for SW620) for four days. Cells were lysed, and protein or mRNA levels tested. All MISSION® shRNAs for FBXL5 (shL5) target the CDS (shL5-1, TRCN4290; shL5-2, TRCN4291; shL5-3, TRC4292; shL5-5, TRC4294), except shL5-4 (TRCN4293) that binds to the 3′-UTR. To further analyse FBXL5 shRNAs, the original vectors were modified removing the puromycin selection cassette and placing GFP in its place, allowing for transfected cells to be sorted to obtain a stable pool of cells. Selected cells were analysed for Snail1 expression by western blot or quantitative (q) real-time (RT)-PCR. Alternatively, to deplete Snail1 from cells, these were transfected with specific synthetic small interfering RNAs (siRNAs) for human SNAIL1 (L-010847-01; from Dharmacon) or an siRNA control.

### Preparation of recombinant proteins

Recombinant proteins were prepared from baculovirus-infected Sf9 cells or *Escherichia coli*. pFastBac baculovirus vectors coding for HA-Cullin1, 6×His-Skp1, Roc/Rbx1, 3×Flag-FBXL5, 6×His-Snail1-HA and GST-Snail1-HA were used to generate recombinant bacmids with the Bac-to-Bac® system (Invitrogen). Isolated bacmids were transfected in Sf9 cells using the Cell Fectin II reagent (Invitrogen) to generate high-titer baculovirus. Infection of Sf9 cells was performed for 2–3 days and cell pellets were snap-frozen and stored at −80°C. Purification of the SCF^FBXL5^ or SCF^FBXL14^ complexes was done after infection of 6xHis-Skp1, HA-Cullin1, Rbx1 and 3xFlag-FBXL5 or FBXL14-HA baculovirus at ratio 1:2:1:1, respectively. Lysis and purification was done essentially as previously described (21). For GST-Snail1-HA purification from infected Sf9 cells or from *E. coli* (GST, GST-Snail1-HA and GST-FBXL5), the protocol was previously described (26). Purification of 3×Flag-FBXL5 was carried out as for GST-Snail1, but using anti-Flag M2 affinity gel (A2220), and elution using the 3×Flag peptide (F4799, both from Sigma) following manufacturer’s instructions. In all cases, buffer exchange after elution was performed using chromatography columns (Micro Bio-Spin® 6, BioRad) and protein stored at −80°C. All purified proteins and complexes were analysed by sodium dodecyl sulphate–polyacrylamide gel electrophoresis and Coomassie staining and quantified using a bovine serum albumin standard and image software analysis (Image Quant, GE Healthcare LifeSciences).

### *In vitro* and *in vivo* ubiquitination

For *in vitro* ubiquitination, typical reactions were composed of 10–40 ng 6×His-Snail1-HA or GST-Snail1-HA, 0.5–2 μg SCF^FBXL5^ or SCF^FBXL14^ (all purified from Sf9 insect cells unless otherwise indicated), 10 μg ubiquitin, 40 ng E1 (6xHis-UBE1, Boston Biochem), 200 ng E3 (UbcH5c, Boston Biochem) in 20 μl reaction buffer containing 10 mM HEPES, pH 7.5, 10 mM KCl, 100 mM NaCl, 1.5 mM MgCl_2_, 1 mM DTT and 25 mM ATP. After 1–2.5 h at 30°C, reactions were stopped with 5× Laemmli buffer and boiled at 95°C for 5 min. For *in vivo* ubiquitination assays using anti-FK2 (Millipore) antibody, cells were transfected and treated with 100 μM FAC and 10 μM MG132 for 4 h. The cytoplasmic fraction was washed out and the pellet resuspended in RIPA buffer (50 mM Tris-HCl, pH 7.5, 150 mM NaCl, 1% sodium deoxycholate, 0.1% sodium dodecyl sulphate, 1 mM ethylenediaminetetraacetic acid, 1% Triton X-100, 2 mM *N*-ethylmaleimide and protease inhibitors) and the protocol continued as previously described (4). Alternatively, *in vivo* ubiquitination assays co-transfecting ubiquitin-HIS plasmid were carried out essentially as described before (4).

### Immunofluorescence

Immunofluorescence was performed as previously described (4), using goat pAb FBXL5 (1:50), mouse mAb Myc hybridoma (1:50) or mouse mAb Snail1 hybridoma (1:1).

### Pull-down assays

HEK293T cells were transfected with the indicated plasmids and pull-down was done as previously described (4). For pull-down assay using *in vitro* purified proteins, 0.2 pmol 3×Flag-FBXL5 were incubated with 2.5 pmol GST or GST-Snail1 (all from baculovirus) as baits. Samples were eluted with 30 µl 2× Laemmli buffer, boiled for 5 min at 95°C and analysed by western blot.

### RNA analyses

RNA was extracted and retro-transcribed as described before (4). Analyses were carried out by qPCR using the LightCycler 480 Real Time PCR System (Roche), with 100 ng per condition and always in triplicate. The primers used were as follows: FBXL5, forward (FW) 5′-CTTACCCAGACTGACATTTCAGATTC-3′, and reverse (RV) 5′-GAAGACTCTGGCAGCAACCAA-3′ [melting temperature (Tm): 53°C]; SNAIL1, FW 5′-GCCTTTCCCACTGTCCTCATC-3′ and RV 5′-TTCCAGCAGCCC TACGACCAG-3′ (Tm: 60°C); FBXL14, FW 5′-TGCCTGTTCCCGGAGCTGCT-3′ and RV 5′- CTTGTGGTAGGCGGCGTCCC-3′ (Tm: 60°C); FBXW5, FW 5′-CGCAG TGCCACAGGCGCCAA-3′ and RV 5′-ACGGGCCCTGCCGTGGTCAT-3′ (Tm: 60°C); FBXW1, FW 5′-CACTCACAGTTTCCAGACAT-3′ and RV 5′-TGCTGAGAGTTTCCGTTGCT-3′ (Tm: 60°C); and hypoxanthine phosphoribosyltransferase 1, FW 5′-GGCCAGACT TTGTTGGATTTG-3′ and RV 5′-TGCGCTCATCTTAGGCTTTGT-3′ (Tm: 60°C).

### Infection, transfection, cell lysis, immunoprecipitation and western blot

Infection of pBabe-Snail1-HA or 6xMyc-FBXL5 was carried out using HEK293 Gag-pol cells (29) transfected with 7.5 μg of the indicated pBabe vector and 2.5 μg of pCMV-VSV-G using polyethylenimine (Polysciences Inc) for 24 h. Two rounds of transduction of the virus were carried out and infected cells were selected with puromycin for 72 h (2.5 µg/ml for RWP-1). Cell transfection, preparation of subcellular fractions, immunoprecipitation and western blot analysis were also performed as described (4).

### Electrophoretic mobility shift assay

Electrophoretic mobility shift assay (EMSA) was performed as described before (22). The ^32^P-labelled probe corresponded to E-box 1 in the E-cadherin promoter (positions −64 to −92) with the following sequence: 5′-GGCTGAGGGTTCACCTGCCGCCACAGCC-3. The mutated E-box 1 used was 5′-GGCTGAGGGTTAACCTACCGCCACAGCC-3′ (mutated bases are underlined).

## RESULTS

### Human F-box shRNA screening in tumoural cell lines identified Snail1 as a target of the ubiquitin ligase FBXL5

**Figure 1.**
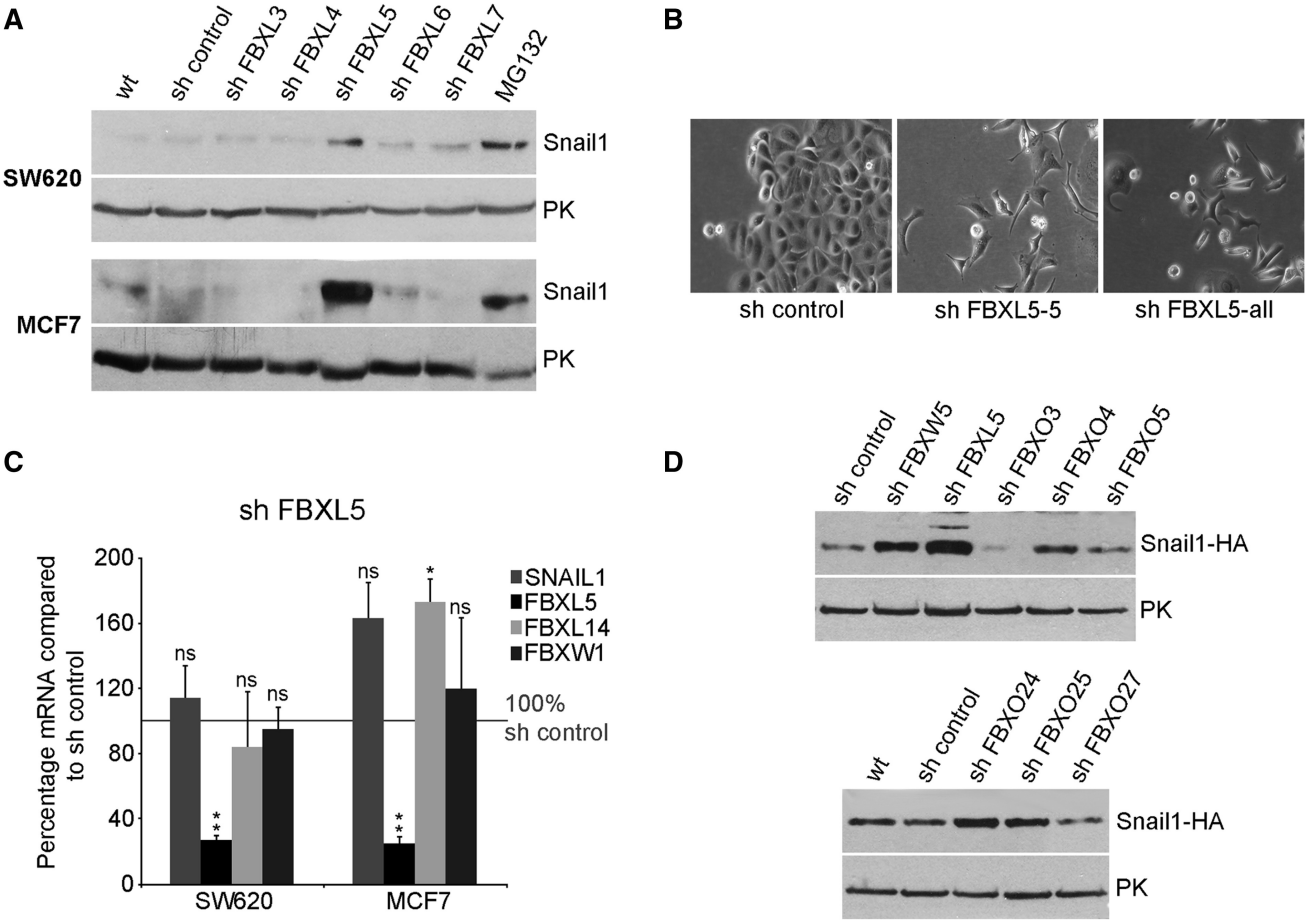
Knock down of FBXL5 increases Snail1 protein levels. (**A**) Snail1 endogenous protein levels were analysed by western blot in SW620 and MCF7 cells infected with shRNA lentiviral vectors targeting different SCF ubiquitin ligases as part of a screening (see also Supplementary Figure S1). Pyruvate kinase (PK) was used as loading control. (**B**) Representative micrographs of MCF7 cells infected with shRNA control, a single FBXL5 shRNA (sh FBXL5-5) or a pool of shRNAs are shown. (**C**) SNAIL1, FBXL5, FBXL14 and FBXW1 mRNA levels were determined by quantitative (q) RT-PCR in SW620 and MCF7 cells after stable expression of the indicated shRNAs and compared with cells transfected with control shRNA. Data are presented as mean ± SD of three independent experiments and referred to the value obtained in control conditions. *P-*values were calculated using unpaired, two-tailed Student’s *t*-test (**P < 0.01; *P < 0.05 or not significant, ‘ns’). (**D**) Putative ubiquitin ligases targeting Snail1 and positive for the first screening were knocked down by shRNA interference in MCF7 cells stably expressing pBabe-Snail1-HA. Snail1-HA protein levels were analysed by western blot.

To identify new F-box proteins that degrade Snail1, we infected SW620 colon cancer cells with a shRNA library targeting 53 human F-box proteins. We chose SW620 cells, as they express moderate Snail1 levels (4). After infection of SW620 with a pool of 4–5 shRNAs against each F-box protein, we analysed Snail1 expression by western blot. We used the proteasome inhibitor MG132 as positive control of Snail1 stabilization. Quantitative (q) RT-PCR analyses revealed a reduction higher than 60% in the RNA levels of several representative F-box proteins (the average reduction was 73% ± 14; *n* = 7). Infection of shRNAs against eight F-box proteins (FBXL5, FBXW5, FBXO3, FBXO4, FBXO5, FBXO24, FBXO25 and FBXO27) upregulated Snail1 protein levels (at least by 2-fold) compared with parental cells or cells infected with a control shRNA (Figure 1A and Supplementary Figure S1A). We repeated the same screening in MCF7 cells, a breast cancer cell line that expresses low Snail1 levels and presents an epithelial phenotype. Only depletion of FBXL5 (Figure 1A) caused a strong accumulation of Snail1. MCF7 cells infected with a pool of shRNAs against FBXL5 (sh FBXL5-pool) or with a single shRNA (sh FBXL5-5) adopted a fibroblastic-like morphology suggesting they had undergone an EMT, whereas cells expressing control shRNA (sh control) maintained the epithelial phenotype (Figure 1B). In both SW620 and MCF7 cells, the FBXL5 shRNA pool promoted a similar downregulation in FBXL5 mRNA without significantly decreasing the levels of FBXL14 and FBXW1, the other described member of this family targeting Snail1 (Figure 1C). SNAIL1 RNA levels were not modified by FBXL5 shRNA in SW620, but modestly increased in MCF7 cells (Figure 1C). To discard that the effect of FBXL5 shRNA on Snail1 was mainly transcriptional in MCF7 cells, we checked whether FBXL5 inhibition affected the stability of exogenously expressed Snail1-HA. We also analysed the effects of shRNA corresponding to other ubiquitin ligases promoting Snail1 upregulation in SW620 cells. As shown in Figure 1D, only infection of sh FBXL5 and, to a minor extent, that of FBXW5, caused an increase in Snail1-HA levels; no stabilization was observed with shRNAs against FBXO3, FBXO5 and FBXO27 and only a modest increase with shRNAs against FBXO4, FBXO24 and FBXO25, suggesting that Snail1 upregulation by depletion of these ubiquitin ligases is indirect. We also verified that FBXL5 and FBXW5 shRNA act specifically on their target genes and were not decreasing the other ubiquitin ligase; as shown in Supplementary Figure S1B the crossed inhibition in SW620 cells was low, whereas in MCF7 the FBXL5 shRNA actually increased the expression of FBXW5 RNA. Finally, the effects of FBXL5 depletion on Snail1 levels were also detected in other cell lines such as RWP-1 (see Supplementary Figure S1C). FBXL5-depletion promoted the acquisition of a more mesenchymal phenotype that was reversed by transfection of an siRNA against SNAIL1, suggesting that Snail1 protein stabilization is responsible for the phenotypic change in these cells (Supplementary Figure S1D). Therefore, taking into consideration the consistent and remarkable effect observed on Snail1 protein stability after FBXL5 depletion in several cell lines we focused our attention on this ubiquitin ligase.

### FBXL5 and Snail1 interact

**Figure 2.**
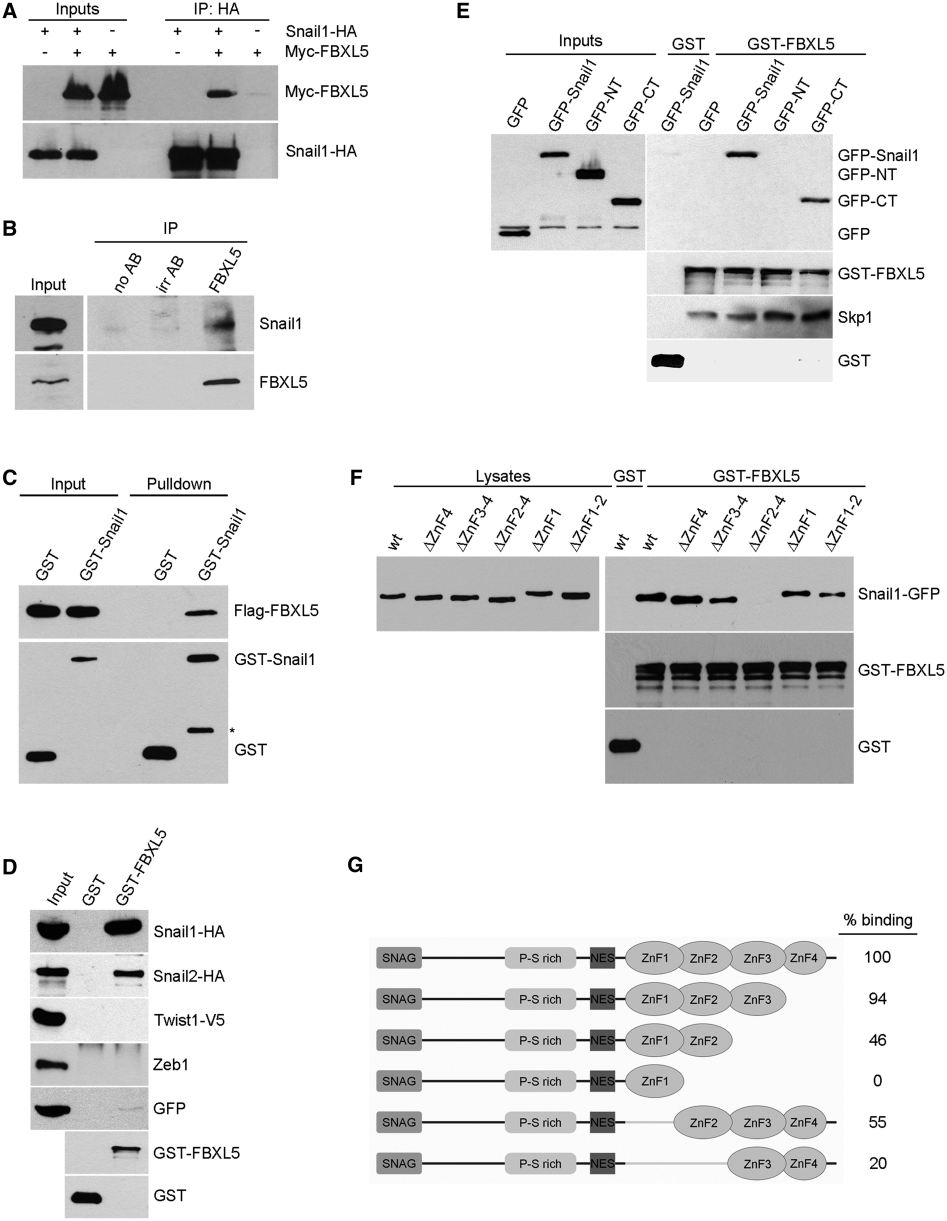
FBXL5 interacts with the C-terminal domain of Snail1. (**A**) HEK293T cells were transfected with control pcDNA3 or pcDNA3-Snail1-HA and Myc-FBXL5 as indicated. Cells were treated with 100 µM FAC (for 6 h) and 10 µM MG132 (for 4 h). Cell extracts were prepared, immunoprecipitated (IP) using a rabbit anti-HA pAb and Myc-FBXL5 or Snail1-HA levels analysed by western blot. (**B**) Endogenous immunoprecipitation of FBXL5 was carried out in RWP-1 cells treated with MG132 and FAC as above using rabbit anti-FBXL5 pAb and an irrelevant antibody (irr AB) or no antibody (no AB) as negative controls. Endogenous Snail1 or FBXL5 (goat pAb) was detected by western blot. (**C**) Pull-down assays were performed using GST-Snail1 and Flag-FBXL5 both purified from Sf9 cells to check whether the interaction between the two proteins is direct. Western blot was used to analyse the results. Flag-FBXL5 was retained by glutathione-sepharose beads when GST-Snail1 and not GST was used as bait. Asterisk indicates a proteolysed fragment of GST-Snail1 observed after experimental incubation conditions. (**D–F**) Similar pull-down assays were carried out with GST or GST-FBXL5 protein and extracts from HEK293T cells expressing Snail1-HA, Snail2-HA, Twist1-V5, Zeb1 or GFP as negative control (D), GFP-Snail1, GFP-NT, GFP-CT or GFP as control (E) or vectors encoding for the full C-terminal domain with the four zinc fingers (ZnF) or deletion mutants (ΔZnF4, ΔZnF3-4, ΔZnF2-4, ΔZnF1 and ΔZnF1-2) (F). Glutathione–sepharose-bound proteins were analysed by western blot. Skp1 binding was also analysed to check the functionality of GST-FBXL5 (E). The results were analysed by western blot using antibodies against the GFP tag (E and F). (**G**) Schematic representation of the deletion mutants used in pull-down analysis to map the site of interaction of Snail1 to FBXL5 (F). The figure also shows the quantification of the percentage of binding from three independent experiments.

If FBXL5 directly modulates Snail1 turnover, both proteins should physically interact. We examined this possibility using tagged proteins exogenously expressed in HEK293T. To maximize FBXL5 expression, cells were treated with FAC, as the stability of FBXL5 is iron dependent (20,21). The proteasome inhibitor MG132 was also added to prevent Snail1 degradation. In these conditions, the two proteins associate, as antibodies against Snail1-HA co-immunoprecipitated Myc-FBXL5 (Figure 2A). We verified the interaction also with endogenous proteins using a goat pAb FBXL5 antibody capable of detecting the protein in cell extracts; the specificity of this antibody was determined by its reactivity against the ectopic gene product (Supplementary Figure S2A) and its sensitivity to FBXL5 shRNA (see later in the text); and also a rabbit FBXL5 pAb capable of immunoprecipitating FBXL5. Snail1 was detected in complexes immunoprecipitated with the FBXL5 antibody and not with an irrelevant antibody (Figure 2B). This association was also verified by pull-down assays using purified recombinant proteins: GST-Snail1 associated with Flag-FBXL5 protein (Figure 2C). Interestingly, the interaction was also observed with Snail2 (Slug) but not with other EMT inducers such as Zeb1 and Twist1 (Figure 2D). This contrasts with FBXL14 that, besides Snail1 and 2, binds and degrades Twist1 and Zeb2 (6). To map the binding region of FBXL5 in Snail1 protein, we used GFP-Snail1 deletion mutants comprising the C-terminal (CT) (GFP-CT: amino acids 152–264) or the N-terminal (NT) (GFP-NT: 1–151) parts of Snail1. Only the CT domain of Snail1 interacted with FBXL5 (Figure 2E). GST-FBXL5 also pulled down endogenous Skp1, as expected for a correctly folded and functional F-box protein. The association with Skp1 was not competed by GFP-Snail1 suggesting that an SCF^FBXL5^-Snail1 degradation complex can be assembled *in vitro* (Figure 2E).

The CT part of Snail1 is involved in DNA binding and nuclear import (30). We further defined the contribution of the four zinc finger domains (ZnF) composing the Snail1 CT in FBXL5 binding. Constructs lacking ZnF4 and therefore comprising amino acids (aa) 236–263 retained full binding, but deletion of both ZnF3 (aa 209–235) and ZnF4 decreased it by >50% (see Figure 2F and a quantification of three independent experiments in Figure 2G). Further deletion of ZnF2 (aa 181–208) completely abolished FBXL5 association. Removal of ZnF1 (aa 150–180) decreased binding by 45%, whereas deletion of both ZnF1 and ZnF2 caused an 80% reduction. These experiments suggest that ZnF2 has a predominant role in FBXL5 binding with contributions of ZnF1 and ZnF3.

### FBXL5 is present in the nucleus

**Figure 3.**
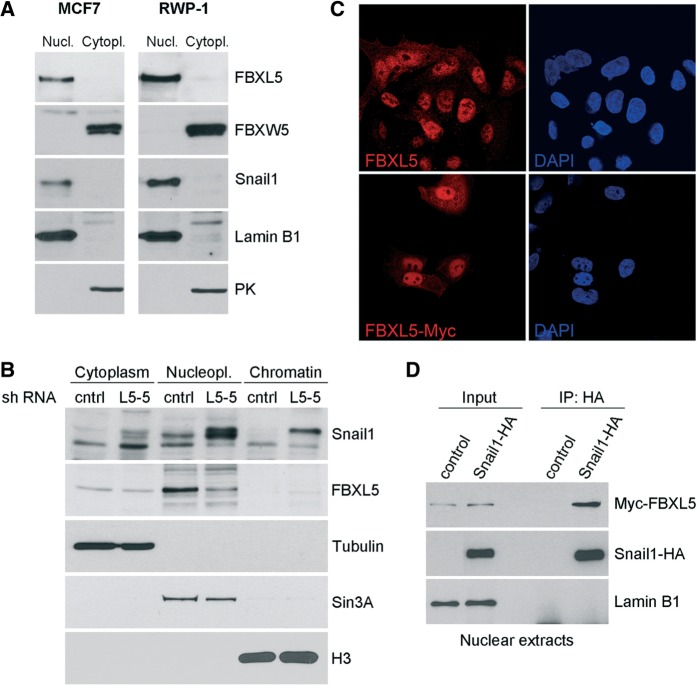
FBXL5 and Snail1 interact in the nucleus. (**A**) Presence of the indicated proteins was determined in nuclear and cytosolic fractions of MCF7 and RWP-1 cells prepared after 4-h treatment with 10 -µM MG132 (4). Pyruvate kinase (PK) and Lamin B1 were used as specific markers of cytosol and nucleus. Distribution of the FBXW5 ubiquitin ligase was also determined. (**B**) MCF7 cells were transfected with plasmids encoding FBXL5 or control shRNAs, and cellular fractions corresponding to the cytoplasm, nucleoplasm and chromatin (DNA-associated proteins) were prepared as indicated and analysed by western blot. Tubulin, Sin3A and histone H3 were used as cytoplasmic, soluble nuclear or chromatin markers, respectively. Note that after fractionation, endogenous Snail1 could be detected as a complex mix of phosphorylated products (26). (**C**) Endogenous (top) or ectopically expressed (bottom) FBXL5 subcellular distribution was determined by immunostaining in MCF7. Cells were treated with 100 μM FAC before fixation. DAPI staining was used to identify nuclei. (**D**) HEK293T cells were transfected with control plasmid, Snail1-HA and Myc-FBXL5 as indicated and treated with 100 µM FAC and 10 µM MG132; the cytoplasmic fraction was washed off and nuclear extracts were used for immunoprecipitation with rabbit anti-HA pAb. Immunocomplexes and inputs were analysed by western blot. Lamin B1 was used as negative control of co-immunoprecipitation.

Analysis of FBXL5 expression showed that it is present in many cell lines both with epithelial and mesenchymal phenotypes (Viñas-Castells *et al.*; unpublished data). Surprisingly, subcellular fractionation of cells revealed that endogenous FBXL5 presents a strong nuclear localization in both MCF7 and RWP-1 cells, unlike the other Snail1-E3 ubiquitin ligases, which have been localized in the cytosol (4,5), and FBXW5, also detected in this compartment (Figure 3A). FBXL5 was present in the nucleoplasm and was not associated to chromatin (Figure 3B). FBXL5 depletion caused a significant upregulation of nuclear Snail1 in MCF7 (Figure 3B) and RWP-1 cells (Supplementary Figure S2B), further validating the screening and antibody specificity. Presence of FBXL5 in the nucleus was also confirmed by confocal immunofluorescence in RWP-1 and MCF7 cells: both the endogenous and ectopic FBXL5 proteins were detected in the nucleus (Figure 3C and Supplementary Figure S2C and D). Endogenous FBXL5 immunofluorescent detection was also decreased by sh FBXL5 (Supplementary Figure S2C). Ectopic FBXL5 was localized both in the nucleus and the cytosol in basal conditions and after iron supplementation with FAC (Supplementary Figure S2E). Interestingly, the nucleus export inhibitor LMB caused the nuclear accumulation of FBXL5 (Supplementary Figure S2E) suggesting that FBXL5 is being actively exported from the nucleus to the cytosol. Finally, we detected a strong interaction between ectopic Snail1-HA and Myc-FBXL5 in the nuclear fraction of HEK293T cells in the presence of the proteasome inhibitor MG132 (Figure 3D). These results strongly suggest that FBXL5 has a predominant role controlling Snail1 abundance and function in the nucleus.

### FBXL5 ubiquitinates Snail1 in the nucleus and causes its degradation

**Figure 4.**
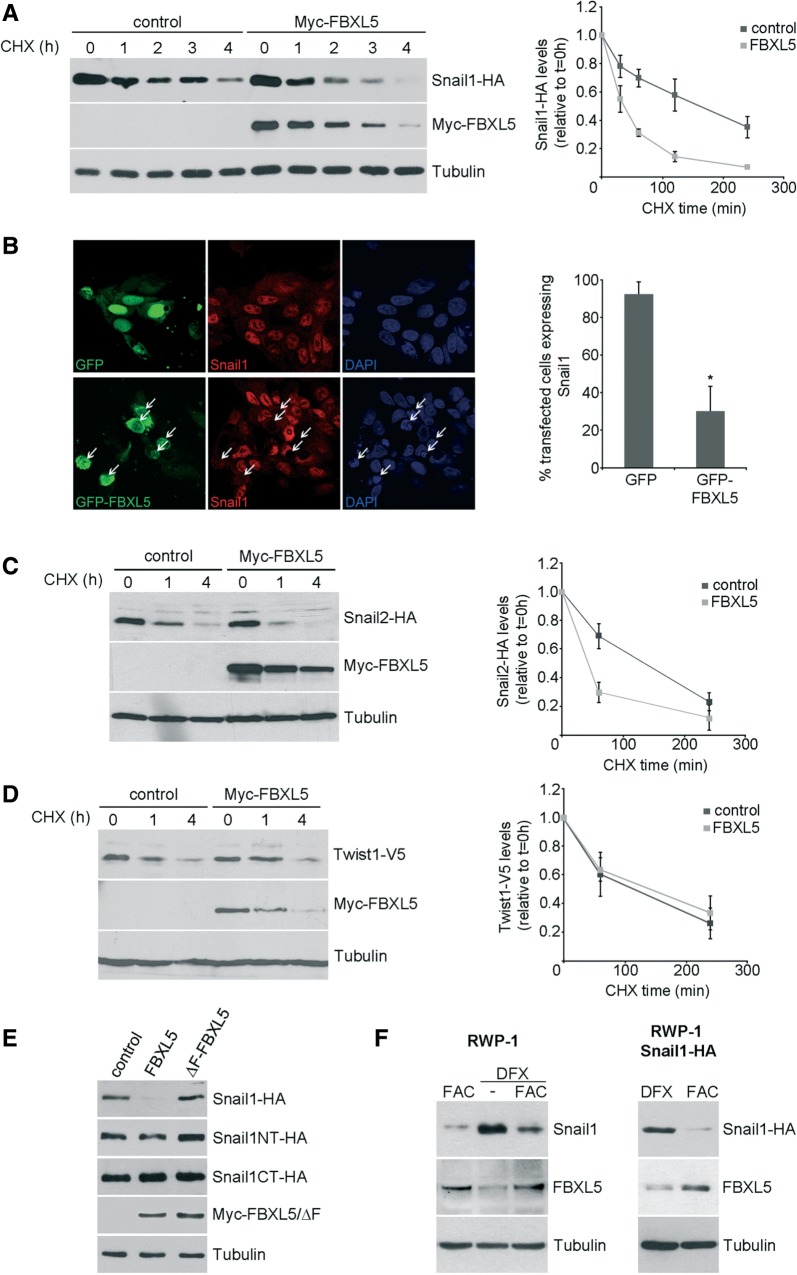
FBXL5 decreases Snail1 half-life. (**A**) HEK293T cells were transfected with Snail1-HA and Myc-FBXL5 or control plasmid for 24 h. Cells were treated for the indicated time with 20 µg/ml CHX and lysed. A representative western blot is shown (left). Right: Snail1-HA levels were analysed by densitometry and normalized for tubulin expression, and the average of the remaining protein ± SD was determined at different times (*n* = 4). (**B**) Immunostaining of RWP-1 transiently transfected with control vector peGFP or GFP-FBXL5 and treated with 100 μM FAC for 4 h (left). DAPI staining was used to identify nuclei. Cells expressing GFP-FBXL5 were indicated with arrows, analysed for positive endogenous Snail1 expression and quantified. At least 100 cells were scored, and results are shown as the mean ± SD (right). Statistical significance was tested using unpaired two-tailed Student’s *t*-test (*n* = 3; *P < 0.05). (**C** and **D**) Snail2, but not Twist1, is degraded by FBXL5. HEK293T cells were transfected with pcDNA3-Snail2-HA (C), pcDNA3-Twist1-V5 (D) and empty plasmid or Myc-FBXL5 for 24 h and treated for the indicated time with 20 μg/ml CHX. The average of the densitometric analyses determining Snail2 or Twist1 levels relative to tubulin (*n* = 3) is shown (right). (**E**) HEK293T cells were transfected with expression vectors encoding Snail1-HA, Snail1NT-HA, Snail1CT-HA and control, Myc-FBXL5 or Myc-ΔF-FBXL5 plasmids. Results were analysed by western blot using HA or Myc antibodies. (**F**) RWP-1 or RWP-1 Snail1-HA cells were treated with 100 μM FAC for 6 h, with 200 μM DFX for 16 h or first with DFX and then with FAC and analysed by western blot.

To determine whether FBXL5 degrades Snail1, we ectopically expressed FBXL5 and quantified Snail1 protein stability after CHX treatment. As shown in Figure 4A, the half-life of Snail1-HA was decreased by 3.6-fold when Myc-FBXL5 was transfected compared with control cells (Figure 4A). To further demonstrate the control of Snail1 expression by FBXL5, we transfected GFP-FBXL5 in RWP-1 cells (Figure 4B). This fusion protein was localized both in the nucleus and in the cytoplasm. Most of the cells transiently transfected with the GFP-FBXL5 construct were depleted of endogenous Snail1 compared with GFP-transfected cells, although ∼30% of FBXL5-expressing cells were still positive for Snail1 expression, presenting resistance to FBXL5 degradation (Figure 4B). We also analysed whether FBXL5 degrades Snail2 and found that this is the case (Figure 4C). In agreement with the results of the pull-down assays, Twist1 protein stability was not affected (Figure 4D). Expression of Snail1-HA full-length protein was downregulated when co-transfected with FBXL5 but not with an inactive FBXL5 deletion mutant lacking the F-box domain (Myc-ΔF-FBXL5) (Figure 4E). FBXL5 was unable to affect the expression of Snail1 NT or CT deletion mutants (Figure 4E), suggesting that the localization of the degron is not in the same domain as the binding site, located in the CT (see Figure 2). This behaviour contrasts with that of the Ppa/FBXL14 ubiquitin ligase, which only needs the NT of Snail1/2 to retain full degradation capability (4,7).

We also investigated whether the manipulation of endogenous iron levels, which control FBXL5 expression, also regulated Snail1 protein levels. Treatment of cells with the iron chelator deferroxamine (DFX) destabilized FBXL5 protein and strongly upregulated both endogenous and exogenous Snail1 in RWP-1 cells (Figure 4F). Reintroduction of iron in RWP-1 by addition of FAC in DFX-treated cells restored FBXL5 levels and abrogated the upregulation of Snail1 (Figure 4F). These results suggest that iron indirectly modifies Snail1 levels through the control of FBXL5 stability.

**Figure 5.**
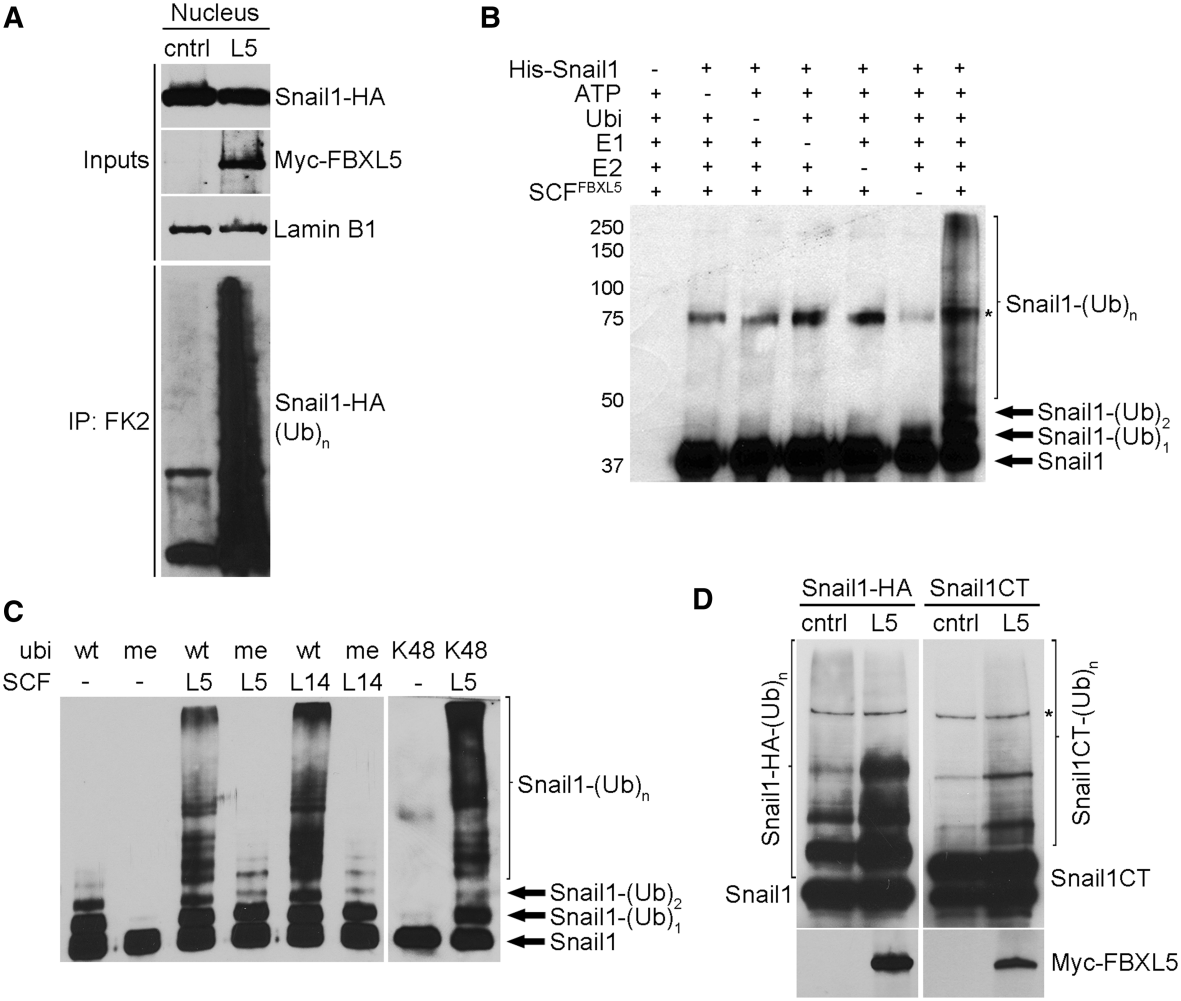
Snail1 is ubiquitinated by the SCF^FBXL5^ complex. (**A**) HEK293T cells were transfected with pcDNA3-Snail1-HA and Myc-FBXL5 (L5) for 24 h and treated with 10 µM MG132 for 3 h. The figure shows the analysis by western blot of immunocomplexes (IP) obtained from nuclear extracts with the mouse anti-ubiquitin FK2 antibody. (**B**) An *in vitro* ubiquitination assay was carried out for 1 h 30 min using Snail1 and SCF^FBXL5^ complex proteins, all recombinant and purified from insect cells. The figure shows the result of an immunoblot performed using the specific Snail1 antibody. The arrows indicate different Snail1 ubiquitination forms (Ub). Asterisk indicates an unspecific band. (**C**) A similar experiment to (B) was carried out using different ubiquitin forms (wt, wild-type ubiquitin; me, methyl ubiquitin; K48, ubiquitin with all lysines mutated to arginine except lysine 48) and SCF^FBXL14^ or SCF^FBXL5^. (**D**) RWP-1 cells were transfected with Snail1-HA or Snail1CT-HA, a His-tagged ubiquitin plasmid and Myc-FBXL5 or a control vector for 24 h, treated with 25 μM MG132 for 4 h and ubiquitinated proteins pulled-down with Ni-NTA beads in denaturing conditions. Modified Snail1 was detected using anti-HA antibodies. Asterisk indicates an unspecific band.

Because FBXL5 is localized in the nucleus, we investigated whether it is capable of ubiquitinating Snail1 in this subcellular compartment. As shown in Figure 5A, co-transfection of FBXL5 increased the amount of polyubiquitinated Snail1 in the nucleus compared with cells transfected with the empty vector. Moreover, analysis of RWP-1 cells co-transfected with Snail1 and FBXL5 and treated with MG132 also showed the presence of a ladder corresponding to modified Snail1, both in whole-cell and nuclear extracts (Supplementary Figure S3A). Finally, incubation of RWP1 cells with LMB, which blocks Snail1 nuclear export (26), did not affect Snail1 polyubiquitination by FBXL5 (Supplementary Figure S3B), further supporting the conclusion that this modification takes place in the nucleus.

To demonstrate that FBXL5 has a direct activity on Snail1, we carried out polyubiquitination reactions *in vitro*. For this we purified Snail1 and the SCF^FBXL5^ complex from Sf9 insect cells infected with different baculoviruses corresponding to Flag-FBXL5, His-Skp1, HA-Cullin1 and Rbx1 (Supplementary Figure S3C). Purified SCF^FBXL5^ ubiquitinated Snail1 only when E1 and E2 enzymes, ubiquitin and ATP were added to the reaction (Figure 5B). The extent of the polyubiquitination reaction obtained with the SCF^FBXL5^ complex was comparable with that achieved with SCF^FBXL14^ (Figure 5C). *In vitro* polyubiquitination took place predominantly through the formation of Lys48-linked ubiquitin chains, as a ubiquitin mutant that has all the Lys except Lys48 mutated to Arg (K48 ubiquitin) was as effective as wild-type (wt) ubiquitin for *in vitro* polyubiquitination of Snail1 (Figure 5C). We also carried out these assays with methyl-ubiquitin (meUbi) that can be conjugated but cannot itself serve as a target for ubiquitination and therefore inhibits elongation of ubiquitin chains. With this reagent, we detected only a discrete pattern of bands corresponding to mono-, bi- and tri-ubiquitinated Snail1, both with FBXL5 and FBXL14 (Figure 5C). This indicated that at least three different lysines are modified and that the ladder obtained with wt ubiquitin comes from polyubiquitination and not from multiubiquitination (monoubiquitination of multiple Lys). Accordingly, mass spectrometry (MS) analysis of Snail1 polyubiquitinated with SCF^FBXL5^ identified Lys85 and 146, both located at the N-terminal part, as being modified (Supplementary Figure S3D and E). Interestingly, we have previously identified Lys146 as one of the residues in Snail1 required for FBXL14- and β-TrCP1-mediated degradation (4). Lys234, in ZnF4 of the CT, was also identified as a target Lys modified by SCF^FBXL5^ (Supplementary Figure S3D and E). We confirmed the ubiquitination of the CT lysine by *in vivo* ubiquitination assays, although Snail1CT was modified to a lower extent than the full-length protein (Figure 5D). In summary, these results demonstrate that Snail1 is ubiquitinated by the SCF^FBXL5^ complex both *in vitro* and *in vivo* and that different lysines located in the N- and C-terminal domains are modified.

### Snail1 ubiquitination by FBXL5 decreases binding to DNA

**Figure 6.**
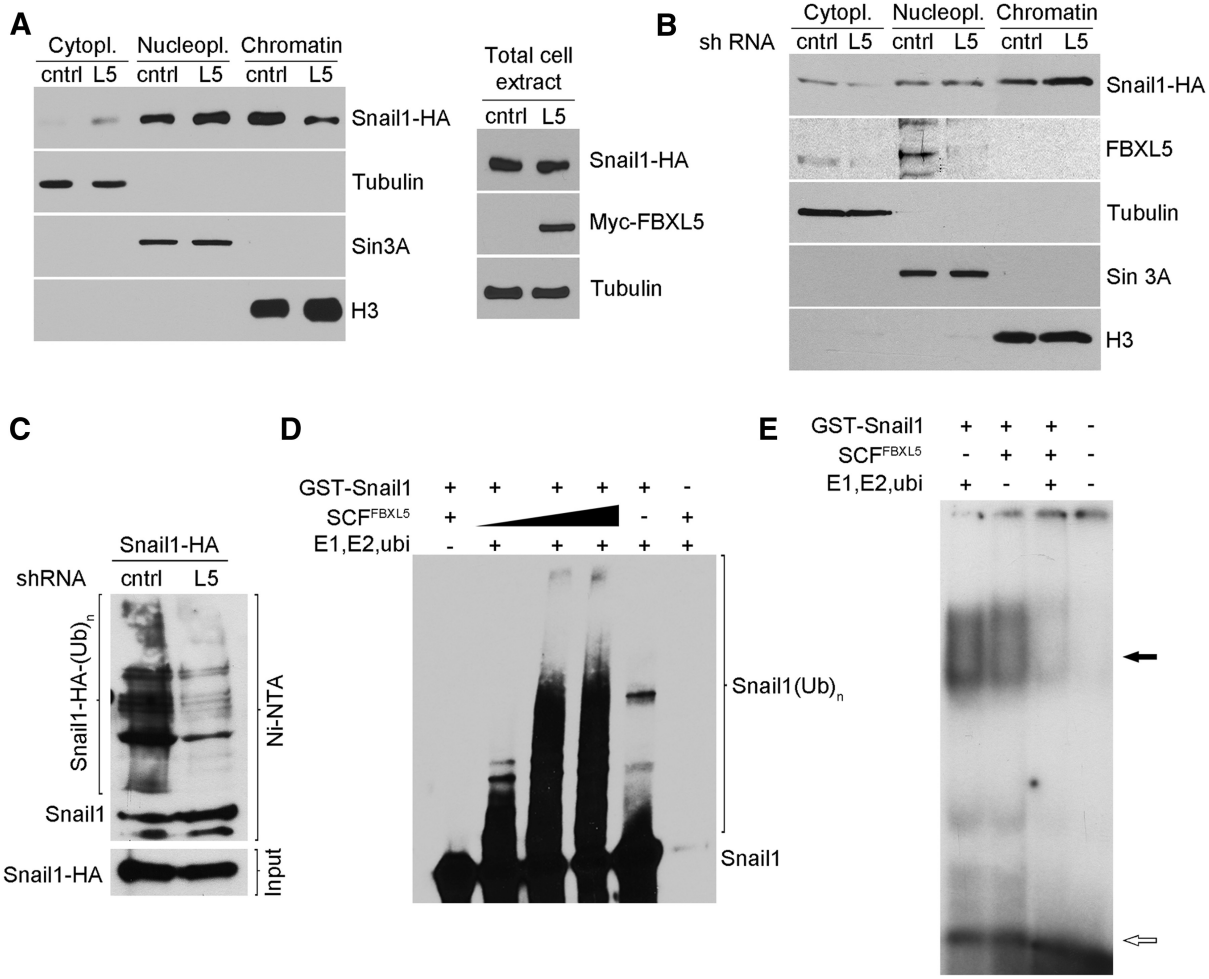
Ubiquitination by FBXL5 prevents Snail1 binding to DNA. (**A**) RWP-1 Snail1-HA cells were transfected with pcDNA3 control (cntrl) or Myc-FBXL5 (L5) for 48 h and treated with 10 µM MG132 and 100 µM FAC for 4 h. Total cell extracts (right) or cytoplasmic, nucleoplasmic and chromatin fractions (left) were obtained and analysed by western blot. Tubulin, Sin3A and H3 expression were determined as markers of the three subcellular fractions. (**B**) RWP-1 Snail1-HA cells were infected with the indicated lentiviral shRNAs and treated as in (A). (**C**) Cells described in (B) were transfected with His-tagged ubiquitin. Polyubiquitinated Snail1 levels were determined as in Figure 5D. (**D** and **E**) *In vitro* ubiquitination was carried out using recombinant GST-Snail1, E1, E2 protein, ubiquitin and different concentrations of the SCF^FBXL5^ complex. Extent of ubiquitination was verified by western blot using Snail1 antibodies (D). Samples showing the highest degree of ubiquitination were used to carry out an EMSA incubating the reaction with a ^32^P-labelled E-cadherin-probe (E). The result shows a representative autoradiography of five different experiments performed. The open arrow labels the free probe and the close arrow labels the shifted band.

Because FBXL5 binds and ubiquitinates the CT part of Snail1, the region required for DNA binding, we wondered whether Snail1-FBXL5 interaction might affect this binding. For this purpose, we used RWP-1 Snail1-HA cells transfected with Myc-FBXL5 when indicated and incubated with MG132 to prevent Snail1 degradation; consequently, in these experimental conditions, total Snail1-HA levels were not modified by FBXL5 (Figure 6A, right). When Snail1 levels were analysed in different subcellular compartments, we observed that FBXL5 caused a significant reduction in Snail1 in the chromatin fraction and an increase in both nucleoplasmic and cytoplasmic fractions (Figure 6A, left), suggesting that FBXL5 displaces or prevents Snail1 interaction with the DNA. Accordingly, depletion of endogenous FBXL5 significantly increased Snail1 bound to chromatin (Figure 6B). This downregulation of FBXL5 was associated to a significant reduction in polyubiquitinated Snail1 (Figure 6C).

The interference in DNA binding was also verified *in vitro* by EMSA, using an oligonucleotide containing an E-box corresponding to one of the Snail1-binding sites in the E-cadherin promoter (22). Snail1 was ubiquitinated *in vitro* using SCF^FBXL5^ and the same reaction carried out in the absence of E1, E2 and ubiquitin was used as control. As expected, no Snail1 polyubiquitination was observed without these components (Figure 6D). Ubiquitination decreased Snail1 binding to DNA, as the intensity of the shifted band was clearly downregulated (Figure 6E). This band was specific, as it was dependent on Snail1 addition, super-shifted with anti-HA antibodies and competed with cold wt but not mutant probe (Supplementary Figure S4). Presence of SCF^FBXL5^ in the EMSA did not substantially decrease the association of Snail1 with the DNA, suggesting that the Snail1 affinity for DNA is much greater than for the ubiquitin ligase.

### Degradation of Snail1 by FBXL5 is prevented by Lats2

**Figure 7.**
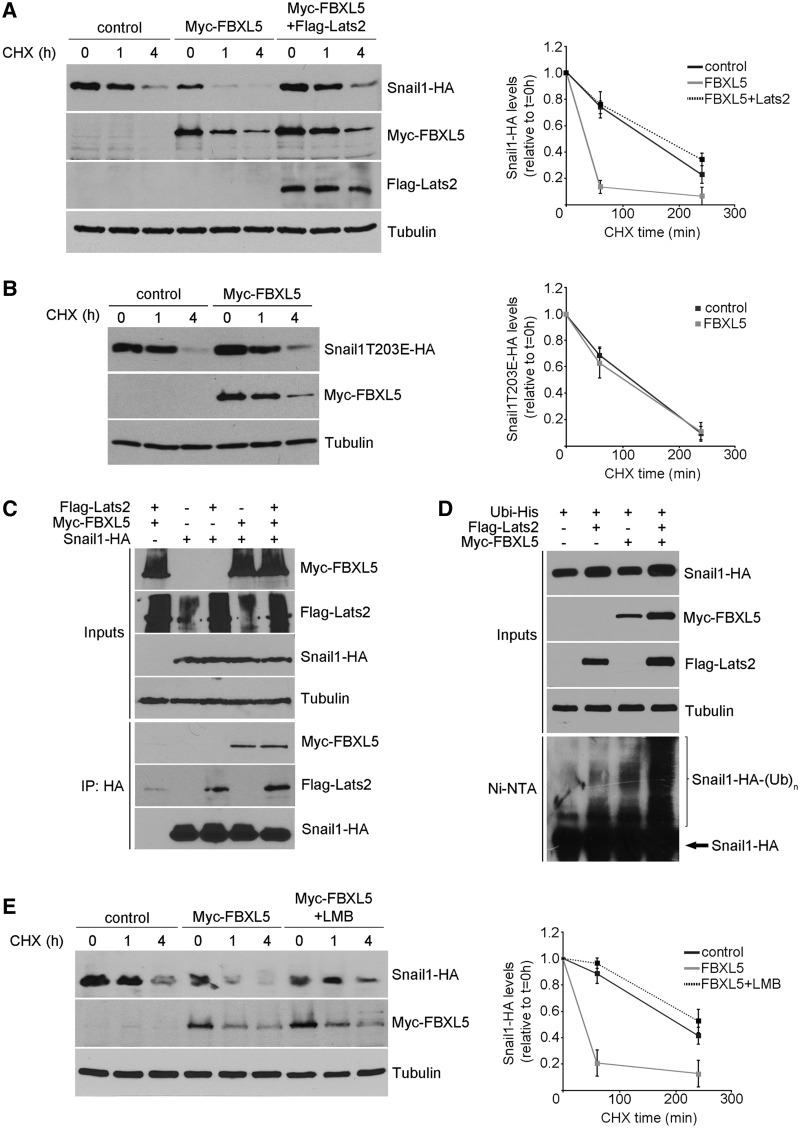
Lats2 prevents Snail1 degradation induced by FBXL5, but not Snail1, polyubiquitination. (**A** and **B**) Snail1-HA (A) or Snail1T203 mutant (B) expression was determined in HEK293T cells at different time points after the addition of CHX in the presence of FBXL5 (A, B) or FBXL5 and Lats2 (A) and compared with control conditions. (**C**) Immunoprecipitation of cell extracts transfected with Flag-Lats2, Myc-FBXL5 and Snail1-HA as indicated and treated with 10 μM MG132 and 100 μM FAC for 4 h using rabbit anti-HA pAb. (**D**) *In vivo* ubiquitination assay performed as previously described in Figure 5D after co-transfection of Snail1-HA, Myc-FBXL5, Flag-Lats2 and ubiquitin-His in RWP-1 cells. Ubiquitinated proteins were purified with Ni-NTA and analysed by western blot. (**E**) CHX time-course experiment performed as in (A) in the presence of the nuclear export inhibitor LMB, added 1 h before CHX addition and maintained during the entire time-course. (A, B, E, right): densitometric analyses showing the quantification of Snail1-HA forms relative to tubulin (*n* = 3).

It has been reported that Snail1 protein can be phosphorylated on Thr203 by Lats2 protein kinase, promoting its stabilization (12). Because this residue is placed in the second zinc finger, which is involved in FBXL5 binding, we checked if Lats2 interfered in Snail1 degradation by this ubiquitin ligase. As shown in Figure 7A, transfection of Lats2 prevented the downregulation of ectopic Snail1 expression induced by FBXL5. We also used a Snail1 mutant (T203E) that mimics Lats2 phosphorylation. Although this mutant did not accumulate in the nucleus as much as the wt form, described to be due to deficient import (12), a significant part of the protein was detected in this compartment after transfection in to HEK293T cells (Supplementary Figure S5A). This Snail1T203 mutant was resistant to FBXL5 degradation (Figure 7B). However, and surprisingly, FBXL5 binding to Snail1 was not affected. As shown in Supplementary Figure S5B, in *in vitro* pull-down assays, GST-FBXL5 interacted with Snail1 T203E, a mutant mimicking phosphorylation, similarly than with the wt protein. To discard that modification of another residue might affect the interaction, we analysed the binding capability of Snail1 purified from Lats2-expressing cells and observed no differences regardless of Lats2 expression (Supplementary Figure S5C). Moreover, in co-immunoprecipitation assays carried out with extracts from cells where degradation was prevented with MG132, we observed that Snail1 and FBXL5 associated to a similar extent in the presence or absence of Lats2 (Figure 7C). Finally, and surprisingly, ubiquitination of Snail1 protein induced by FBXL5 was not prevented but enhanced in Lats2-transfected cells (Figure 7D). These results suggest that despite inhibiting the degradation of the Snail1 protein, Lats2 does not prevent its interaction and ubiquitination by FBXL5.

Lats2 stabilization of Snail1 has been associated to Snail1 retention in the nucleus (12). It is plausible to think that, although Snail1 is ubiquitinated by FBXL5 in the nucleus, its degradation happens in the cytosol and requires the export of the ubiquitinated protein. Lats2 may be preventing the nuclear exit, and thus the degradation by FBXL5. To verify this hypothesis, we determined the effect of LMB, a nuclear export inhibitor, in Snail1 degradation by FBXL5. As previously shown in Supplementary Figure S3B, LMB did not affect Snail1 polyubiquitination caused by FBXL5; however, it greatly prevented FBXL5 effect on Snail1 half-life (Figure 7E), indicating that the traffic to the cytosol is required to complete the destabilization of Snail1 protein. In accordance with this requirement, a Snail1 form deficient in nuclear export owing to the mutation of the nuclear export sequence (NES) [Snail1LA (26)] was resistant to degradation by FBXL5 (Supplementary Figure S5D) while it was ubiquitinated by FBXL5 with a similar efficiency as the wt protein (Supplementary Figure S5E).

### Snail1 stabilization by γ-irradiation is associated with FBXL5 down-modulation

**Figure 8.**
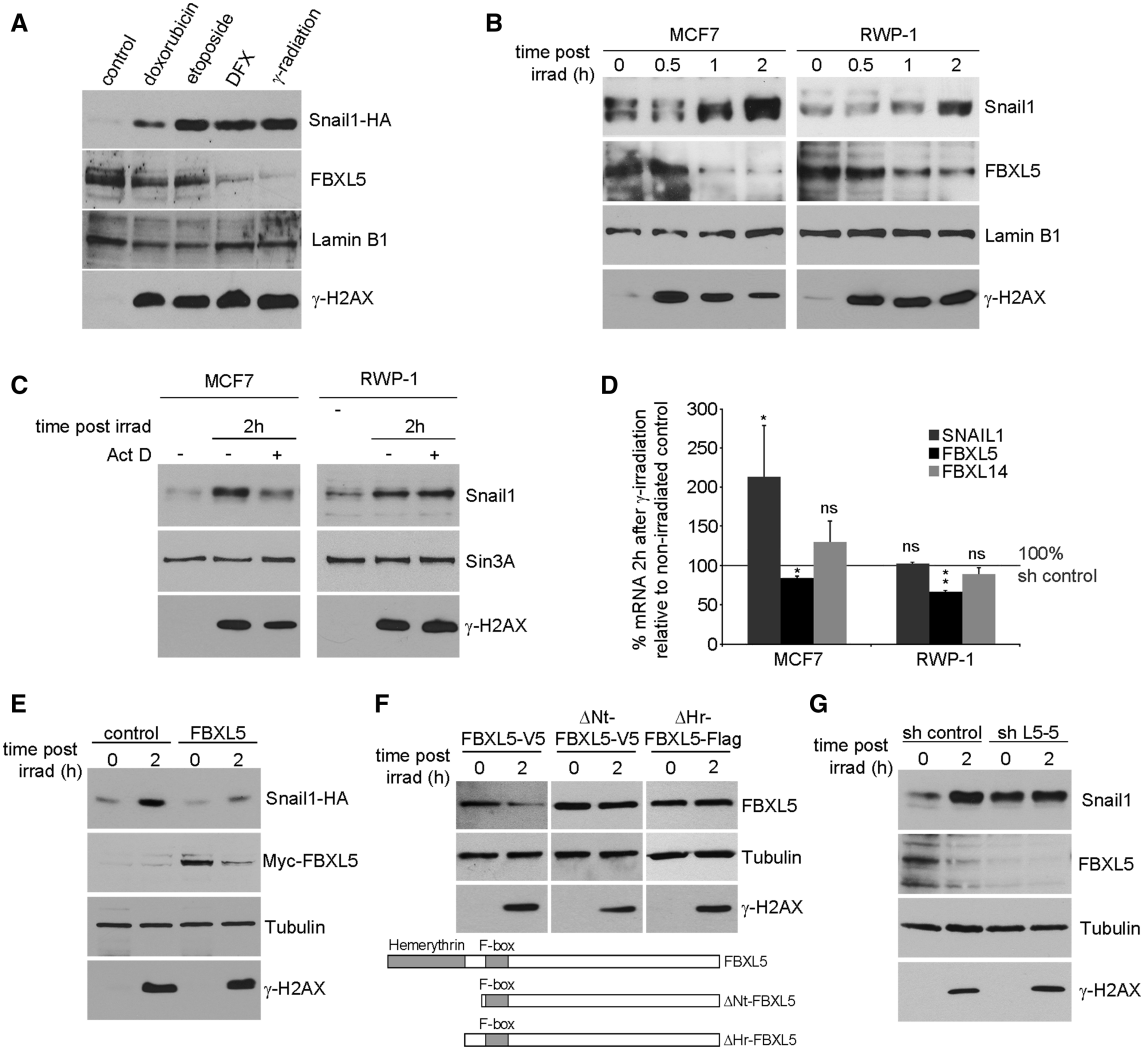
Ionizing radiation promotes FBXL5 degradation and post-translational stabilization of Snail1. (**A**) RWP-1 cells stably expressing Snail1-HA were subjected to different insults: 10 μM doxorubicin for 4 h, 10 μM etoposide for 4 h, 100 μM DFX for 16 h, γ-irradiation (20 Gy, lysis was 2 h after irradiation) and Snail1-HA and FBXL5 endogenous levels analysed by western blot in enriched nuclear extracts. Lamin B1 expression was used as loading control. (**B**) MCF7 and RWP-1 nuclear extracts were prepared at different times after 20 Gy irradiation and analysed by western blot. (**C**) The indicated cells were treated with 2.5 µg/ml Act D for 1 h, subjected to 20 Gy γ-irradiation, lysed after 2 h and analysed by immunoblotting. (**D**) mRNA from MCF7 and RWP-1 cells was obtained 2 h after 20 Gy irradiation and analysed by qRT-PCR to determine the expression of the indicated genes. Data are presented as average ± SD. and referred to the value obtained in control conditions. *P-*values were calculated using unpaired two-tailed Student’s *t*-test (*n* = 3 for all cases and *n* = 4 for FBXL14 quantification; **P < 0.01; *P < 0.05 or not significant, ‘ns’). (**E**) RWP-1 Snail1-HA cells were stably infected with pBabe control or Myc-FBXL5 vectors, irradiated with 20 Gy and lysed after 2 h. Analysis was performed by western blot. (**F**) RWP-1 cells were transfected with the indicated FBXL5 deletion mutants, irradiated and analysed as in (E). (**G**) RWP-1 cells stably expressing pLKO-GFP sh control or sh FBXL5-5 (sh L5-5) vectors were subjected to 20 Gy ionizing radiation and lysed after 2 h. Protein levels were analysed by western blot. In (A, B, C, E, F and G), phosphorylation of H2AX on S139 (γ-H2AX) was used as positive control for DNA damage.

Snail1 stability increases after several types of cellular stress such as DNA damage or hypoxia (4). We checked the implication of FBXL5 in the stress-dependent Snail1 stabilization by analysing the effect of a panel of different insults (doxorubicin, etoposide, deferroxamine or γ-irradiation) on FBXL5 protein levels. Although all these insults increased Snail1-HA protein levels (Figure 8A), only iron-depletion by deferroxamine and γ-irradiation (IR) elicited a strong downregulation of FBXL5. A time-course analysis showed that 1 or 2 h after IR, Snail1 protein levels were increased in MCF7 and RWP-1 cells (Figure 8B). In RWP-1 cells, this stimulation was mainly post-translational, as it remained unaffected by the addition of the transcription inhibitor Act D, whereas in MCF7 the inhibition by this drug was only partial (Figure 8C). Accordingly, SNAIL1 mRNA levels were not affected by IR in RWP-1 and were only slightly stimulated in MCF7 cells, indicating that increased transcription does not significantly contribute to Snail1 upregulation (Figure 8D). The post-translational stabilization of the transcription factor by IR was confirmed in RWP-1 Snail1-HA cells by the increased expression of the protein (Figure 8E). Concomitant to the Snail1 increase on IR, FBXL5 protein was downregulated in both cell lines (Figure 8B). Furthermore, infection of Myc-FBXL5 almost totally abolished the Snail1-HA stabilization caused by irradiation (Figure 8E). Downregulation of FBXL5 by IR was associated with a slight decrease in FBXL5 mRNA (20–40% depending on the cell line; Figure 8D) and a destabilization of FBXL5 protein, as infected Myc-FBXL5 levels were also reduced by IR, suggesting that downregulation of this protein is post-translational (Figure 8E). Interestingly, downregulation of FBXL5 protein depends on the hemerythrin domain (Hr), as deletion mutants lacking this domain (ΔHr) or the complete N-terminal region (ΔNt) were not sensitive to IR (Figure 8F). Finally, as expected, depletion of FBXL5 by shRNA L5-5 promoted a potent Snail1 upregulation that was similar to that obtained after IR in control cells (Figure 8G). Once FBXL5 was depleted, Snail1 levels were no longer affected by IR. These experiments suggest that FBXL5 levels are decreased by IR causing a major stabilization of Snail1 in tumour cell lines.

## DISCUSSION

Two ubiquitin ligases controlling Snail1 protein stability in eukaryotic cells have been previously identified: SCF^β-TrCP1^, that acts in the cytoplasm, dependent on previous GSK-3β phosphorylation and negatively regulated by the Wnt pathway (18); and SCF^Ppa/FBXL14^, also present in the cytosol, downregulated in hypoxia and additionally targeting Snail2, Twist1 and Zeb2 for degradation (4,6). Our shRNA screening of F-box proteins has identified FBXL5 as a novel ubiquitin ligase controlling Snail1 protein stability. FBXL5 was initially characterized as a protein controlling iron homeostasis (20,21); in fact, mice deficient in FBXL5 die *in utero* owing to impaired IRP2 degradation, which leads to iron accumulation (31).

Here we describe that FBXL5 is mainly present in the nucleus, although when over-expressed or stabilized by supplementing the cell culture with iron it is also detected in the cytoplasm. The nuclear localization of FBXL5 was further supported by experiments using the nuclear export inhibitor LMB that promoted a significant increase in accumulation of nuclear FBXL5. This sensitivity to LMB suggests that FBXL5 is exported from the nucleus through the action of Crm1. It is intriguing that two of the substrates previously described for this ubiquitin ligase, IRP1 and IRP2, are cytoplasmic proteins, indicating that FBXL5 is also functional in the cytosol (32). It is possible that two pools of FBXL5 co-exist inside the cell: one cytoplasmic and active in IRP2 degradation; another one, nuclear and controlling Snail1 ubiquitination. Although both pools present similar sensitivity to iron, the fact that the localization of this ubiquitin ligase is mainly nuclear suggests that the cytoplasmic substrates might be more dependent on iron, as depletion of this metal would promote a decrease in FBXL5 levels below the active threshold, preferentially in the cytoplasmic subcellular compartment.

The FBXL5 binding sequence in Snail1 has been mapped to the carboxy-terminal half of the protein (Snail1CT), mainly to ZnF2, although ZnF1 and ZnF3 are also necessary to achieve a full binding capacity. This Snail1 CT domain is well conserved among the different orthologues (33). Accordingly, the interaction of FBXL5 was also observed with Snail2, but not with Twist1 or Zeb1, correlating with both Snail1 and Snail2 half-lives being decreased by FBXL5 but not that of Twist1. This differs from the Ppa/FBXL14 ubiquitin ligase that promotes degradation of Snail1 and Snail2 and also that of Twist1 and Zeb2 (6). These findings have suggested a general role for FBXL14 in controlling EMT mediators and indicate a more specific activity of FBXL5.

Previous reports have shown that the NT, part of both Snail1 and Snail2, controls protein degradation. Accordingly, the β-TrCP1 and FBXL14 degron sequences in Snail1 are located in the NT (4,5). In the case of FBXL5, both Snail1 NT and CT are required for degradation; although the interaction with the ubiquitin ligase takes place through the CT, it is likely that the NT contains the main lysine residues required for degradation. This hypothesis is supported by *in vitro* ubiquitination data showing that the SCF^FBXL5^ complex modifies Lys85 and Lys146, both placed in the NT, as well as Lys234, which is located in the CT. However, Lys234 seems to be modified to a lower extent and may not participate in degradation. On the other hand, Lys146 has been shown to be involved in both β-TrCP1 and FBXL14 degradation (4). It is likely that this lysine is the main substrate for K48-linked polyubiquitination in Snail1.

Because FBXL5 binds the Snail1 CT domain, our initial working hypothesis supposed that the association of the ubiquitin ligase might be impaired by Lats2 kinase through phosphorylation of Snail1 on Thr203 (12), a modification located close to the FBXL5 binding site. As shown here, Lats2 abolishes Snail1 degradation by FBXL5 but does not decrease the association of both proteins *in vivo* or *in vitro*. Moreover, Lats2 does not prevent Snail1 polyubiquitination by FBXL5. These results have several relevant implications. First, they suggest that polyubiquitinated Snail1 is not targeted for degradation in the nucleus, and conditions preventing nuclear export, such as phosphorylation by Lats2, block degradation by FBXL5. We corroborated this hypothesis using LMB and the Snail1LA mutant, unable to be exported due to the alteration of the NES sequence (26). Although Snail1 was polyubiquitinated by FBXL5, in both conditions it was not degraded, further supporting the hypothesis that nuclear export is required for efficient proteolysis of Snail1. It is likely that, if not exported and degraded, FBXL5 action is antagonized by deubiquitinases that may also control EMT (34).

Another interesting consequence refers to Lats2 action. A plausible mechanism to explain its effect considers that phosphorylation on Thr203 in Snail1 recruits a chaperone protein that prevents Crm1 binding and therefore, Snail1 export. In accordance with this possibility, Snail1 protein stability is enhanced by its interaction with two nuclear chaperones, HSP27 and HSP90 (14,35); at least in the case of HSP90, the association is dependent on the previous phosphorylation of Ser100 in Snail1 protein. It remains to be established if the modification on Thr203 facilitates the interaction with HSP27 or another yet uncharacterized chaperone. Besides polyubiquitinating Snail1 NT lysines, FBXL5 also modifies Lys234, placed in the C-terminal domain. Although we cannot discard the involvement of other Lys, it is likely that modification of this residue is responsible for the lower binding to DNA observed for FBXL5-polyubiquitinated Snail1. According to our model, the decreased interaction with the target promoters, together with the unfolding of the molecule exposing the NES, would facilitate Snail1 nuclear export.

FBXL5 is expressed in different cancer cell lines. We did not observe an inverse correlation between the levels of FBXL5 and Snail1, suggesting that mechanisms other than those modulating its expression control the activity of the ligase, for instance, that dependent on Lats2 kinase. Moreover, FBXL5 expression is decreased by some conditions that induce Snail1 stabilization. Besides being destabilized by iron depletion, FBXL5 is potently downregulated after IR, a cellular stress condition promoting EMT (36); this decrease takes place concomitantly with Snail1 stabilization. The downregulation of FBXL5 by IR depends on a reduction of mRNA and a destabilization of the protein that is mediated by the same hemerythrin domain that confers sensitivity to iron. Accordingly, our current model for the regulation of Snail1 protein stability suggests that in the absence of cellular stress Snail1 levels are maintained low in epithelial cells through the coordinated effects of the cytoplasmic F-box proteins β-TrCP1 (FBXW1) and FBXL14, the nuclear FBXL5, and maybe other ubiquitin ligases. This multiple action explains why FBXL5−/−IRP2−/− mice embryos are viable without any apparent Snail1-dependent phenotype (31). However, it is possible that these animals may be specially sensitive to conditions down-regulating FBXL14, such as hypoxia (4), as in the absence of FBXL5, Snail1 should be increased to higher levels. After DNA damage, the combined activation of ATM, which confines Snail1 to the nucleus (14), and the downregulation of the FBXL5 facilitate Snail1 upregulation and EMT. Our results also demonstrate that Snail1 protein levels are rapidly upregulated in response to cellular stress, suggesting that Snail1 and EMT work, likewise to the title of a recent review, as the ultimate survival mechanism of cancer cells (37).

## SUPPLEMENTARY DATA

Supplementary Data are available at NAR Online, including [38–40].

## FUNDING

Fundación Científica de la Asociación Española contra el Cáncer, the Ministerio de Ciencia y Tecnología [SAF2010-16089] and Fundació La Marató de TV3 (to A.G.H.); The authors also acknowledge support from ISCIII/FEDER [RD06/0020/0040] and Generalitat de Catalunya [2009SGR867]; a predoctoral fellowship from ISCIII (to R.V.-C.). Funding for open access charge: Ministerio de Ciencia y Tecnología [SAF2010-16089].

*Conflict of interest statement*. None declared.

## Supplementary Material

Supplementary Data
