# Correction to ‘Nuclear ubiquitination by FBXL5 modulates Snail1 DNA binding and stability’

**DOI:** 10.1093/nar/gkag263

**Published:** 2026-03-17

This is a correction to: Rosa Viñas-Castells, Álex Frías, Estefanía Robles-Lanuza, Kun Zhang, Gregory D. Longmore, Antonio García de Herreros, Víctor M. Díaz, Nuclear ubiquitination by FBXL5 modulates Snail1 DNA binding and stability, Nucleic Acids Research, Volume 42, Issue 2, 1 January 2014, Pages 1079–1094, https://doi.org/10.1093/nar/gkt935

In December 2025, concerns were raised on PubPeer (https://pubpeer.com/publications/8125E2B29BC285753A3BF08B607988) about the potential duplication of the Sin3A bands in Figures 6A and 6B, as well as a possible alteration of the background in the Snail1–HA panel of Figure 6B that could indicate a composite image. The authors have reviewed these issues and now wish to provide a corrected version of Figure 6.

The authors confirmed that the Sin3A bands in Figures 6A and 6B were duplicated during image assembly. They have provided all the blots corresponding to these two figures, including the one corresponding to Sin3A of Figure 6A. For Figure 6B the authors provided a different contemporaneous Sin3A blot confirming the results: Sin3a is present specifically in the NE fraction without differences between shCtl and shL5 conditions. A similar result (no change in Sin3a upon L5 downmodulation) is also shown in other figures of this article (Figures 3B and S2B).

The authors have provided the journal with the original autoradiogram demonstrating that the Snail1–HA panel of Figure 6B is not a composite image.

The original data is included in the Supplementary Information accompanying this correction notice. A revised version of Figure 6, with a corrected panel B, is presented below.

This correction does not affect the results, discussion and conclusions presented in the article. These details have been corrected only in this correction notice to preserve the published version of record.


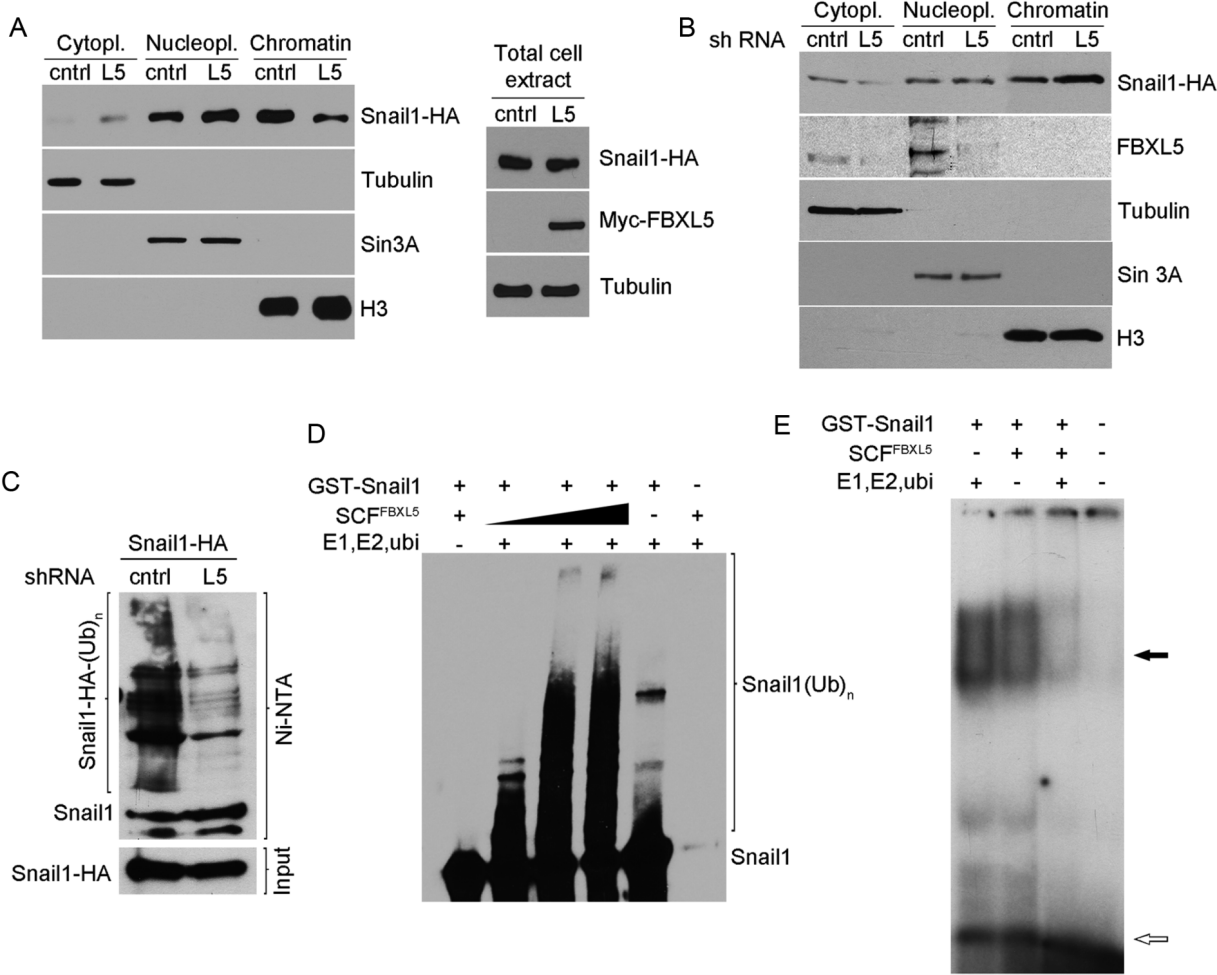


New Figure 6.

## Supplementary Material

gkag263_Supplemental_File

