# Supplementary material for: Nuclear ubiquitination by FBXL5 modulates Snail1 DNA binding and stability
Source: Nucleic Acids Res. 2013 Oct 23;42(2):1079–94. doi: 10.1093/nar/gkt935 (PMC3902928; doi:10.1093/nar/gkt935)
Supplement: Supplementary Data [file supp_42_2_1079__index.html]

Nuclear ubiquitination by FBXL5 modulates Snail1 DNA binding and stability — Nuclear ubiquitination by FBXL5 modulates Snail1 DNA binding and stability — Supplementary Data 

# Nuclear ubiquitination by FBXL5 modulates Snail1 DNA binding and stability

## Supplementary Data

files

**Files in this Data Supplement:**

- Supplementary Data - pdf file
