# Supplementary material for: Nuclear ubiquitination by FBXL5 modulates Snail1 DNA binding and stability
Source: Nucleic Acids Res. 2013 Oct 23;42(2):1079–94. doi: 10.1093/nar/gkt935 (PMC3902928; doi:10.1093/nar/gkt935)
Supplement: Supplementary Data [file supp_gkt935_nar-01455-v-2013-File010.pdf]

Supplementary Information

Nuclear ubiquitination by FBXL5 modulates Snail1 DNA binding and stability

Rosa Viñas-Castells, Álex Frías, Estefanía Robles-Lanuza, Kun Zhang, Gregory D. Longmore, Antonio García de Herreros, and Víctor M. Díaz.

Supplementary material and methods

Mass Spectrometry analysis

For sample preparation, gel bands were destained, dehydrated and digested with trypsin (Promega) and tryptic peptides extracted and cleaned up as previously described (38). Peptide mixes were analysed using LTQ-Orbitrap Velos mass spectrometer (Thermo Fisher Scientific) coupled to an EasyLC (Thermo Fisher Scientific). Peptides were loaded directly onto the analytical column (flow rate of 1.5-2 µl/min) and separated by reversed-phase chromatography using a 12 cm column (inner diameter of 75 µm) packed with 5 µm C18 particles (Nikkyo Technos Co.) .

The mass spectrometer was operated in positive ionization mode with nanospray voltage set at 2.2 kV and source temperature at 250°C. Ultramark 1621 for the FT mass analyser was used for external calibration prior the analyses. Internal calibration was performed using background polysiloxane ion signal at m/z 445.1200. The instrument was operated in DDA mode and full MS scans with one micro scan at resolution of 60,000 over a mass range of m/z 250-2,000 with detection in the Orbitrap. Auto gain control (AGC) was set to 1e6, dynamic exclusion (60s) and the charge state filter disqualifying singly charged peptides was activated. Following each survey scan the top twenty most intense ions with multiple charged ions above a threshold ion count of 5,000 were selected for fragmentation at normalized collision energy of 35%. Fragment ion spectra produced via collision-induced dissociation (CID) were acquired in the linear ion trap, AGC was set to 5e4, isolation window of 2.0 m/z, activation time of 0.1 ms and maximum injection time of 100 ms was used. All data were acquired with Xcalibur software v2.2.

Data Analysis was performed using Proteome Discoverer software suite (v1.3.0.339, Thermo Fisher Scientific) and the Mascot search engine (v2.3, Matrix Science (39) for peptide identification, as previously described (40). A precursor ion mass tolerance of 7 ppm at the MS1 level was used, and up to three miscleavages for trypsin were allowed. The fragment ion mass tolerance was set to 0.5 Da. Ubiquitination (glycine-glycine) at lysines was defined as variable modification. Carbamidomethylation on cysteines was set as a fixed modification. The identified peptides were filtered using a Mascot Ion Score of 20.

Supplementary References

38. Rappsilber, J., Mann, M. and Ishihama, Y. (2007) Protocol for micro-purification, enrichment, pre-fractionation and storage of peptides for proteomics using StageTips. *Nat Protoc*, **2**, 1896-1906.

39. Perkins, D.N., Pappin, D.J., Creasy, D.M. and Cottrell, J.S. (1999) Probability-based protein identification by searching sequence databases using mass spectrometry data. *Electrophoresis*, **20**, 3551-3567.

40. Bunkenborg, J., Garcia, G.E., Paz, M.I., Andersen, J.S. and Molina, H. (2010) The minotaur proteome: avoiding cross-species identifications deriving from bovine serum in cell culture models. *Proteomics*, **10**, 3040-3044.

Supplemental Figure Legends

Supplementary Figure S1. Screening of SCF E3 ubiquitin ligases targeting Snail1 stability. (**A**) Snail1 endogenous protein levels were analysed in SW620 cell line after stable infection of shRNA lentiviral vectors corresponding to SCF ubiquitin ligases. The most relevant Western Blots corresponding to putative candidates are shown. Pyruvate kinase (PK) was determined as loading control. (**B**) mRNA levels of the indicated ubiquitin ligases were determined by quantitative (q) RT-PCR using RNA from cells stably infected with FBXL5 or FBXW5 shRNAs. Data are presented as mean ± s.d. of three independent experiments and referred to the value obtained in control conditions. (**C**) Snail1 endogenous protein levels were increased in RWP-1 cell line after stable infection with sh FBXL5. Transfection of SNAIL1 siRNA for 72 h abolished the upregulation of the transcription factor. (**D**) Representative micrographs of RWP-1 cells infected with shRNA control or a pool of FBXL5 shRNAs and transfected during 72 h as in (C) with control or SNAIL1 siRNAs at low or high cell densities.

Supplementary Figure S2. FBXL5 is a nuclear ubiquitin ligase. (**A**) Validation of FBXL5 antibody used in Western Blot detection and immunofluorescence. HEK293T cells were transiently transfected for 48 h with increasing amounts of pCDNA3-6xMyc-FBXL5 plasmid, lysed and analysed by Western Blot using antibodies against FBXL5 (goat pAb) or the Myc tag. (**B**) RWP-1 cells were stably transfected with pLKO-GFP sh control or sh FBXL5-5 vector and endogenous Snail1 and FBXL5 levels were analysed by Western Blot after sub-fractionation of cell extracts. Tubulin, Sin3A and H3 antibodies were used as cytoplasmic, nucleoplasmic or chromatin loading controls, respectively. (**C-E**) Immunodetection of FBXL5 in MCF-7 (C, E) or RWP-1 cells (D) infected with sh control or sh FBXL5 (C) or transfected with pcDNA3-6xMyc-FBXL5 and treated with 100 µM FAC for 4 h or 5 ng/ml LMB for 2 h (E). DAPI staining was used to identify nuclei in all the panels.

Supplementary Figure S3. Snail1 is ubiquitinated by FBXL5. (**A**) RWP-1 cells were transfected with plasmids encoding Snail1-HA, and empty plasmid or 6xMyc-FBXL5 for 48 h, treated with 100 μM FAC and 10 μM MG132 for 4 h before lysis, and analysed by immunoblotting using anti-HA antibodies. (**B**) *In vivo* ubiquitination of Snail1 in RWP-1. GFP-Snail1-HA and ubiquitin-His were transfected together with FBXL5 or empty vector and treated with LMB when indicated. All samples were treated with 10 μM MG132 prior to lysis. (**C**) Coomassie staining of the purified SCF^{FBXL5} complex used for *in vitro* ubiquitination assays. The left panel is a 12% acrylamide gel whereas the right panel corresponds to the upper part of a 7.5% gel to obtain better resolution of the HA-Cullin1/Flag-FBXL5 proteins. (**D**) Colloidal blue staining of a polyacrylamide gel containing the *in vitro* ubiquitinated Snail1 proteins obtained after a 2 h 30 min reaction. The square box indicates the part of the gel used for MS analysis. (**E**) Output result from MS analysis showing statistically significant peptides obtained presenting the Gly-Gly modification in the indicated lysines of Snail1. Two *in vitro* reactions were used for each analysis. Representative results from two independent experiments are shown.

Supplementary Figure S4. GST-Snail1 binds E-box 1 in the E-cadherin promoter. Increasing amounts of purified GST-Snail1-HA protein from baculovirus were bound to ^{32}P-E-cadherin-probe corresponding to E-box 1 (arrow). Binding was competed with cold wild-type (wt) or mutated (mut) probe, or supplemented with HA or control (IgG) antibodies. The super-shifted band is labelled with a double arrow and the free probe with an open arrow. The results of a representative autoradiography are shown.

Supplementary Figure S5. Snail1 degradation by FBXL5 is dependent on nuclear export. (**A-C**) Lats2 does not prevent FBXL5 binding to Snail1. (A) Western Blot showing the relative distribution of a Snail1 mutant mimicking Lats2 phosphorylation (T203E) compared with the wild-type form in nuclear (NE) and cytoplasmic extracts (CE). (B) A pull-down analysis was performed as described in Fig. 2D with different Snail1 mutants mimicking Lats2 phosphorylation (T203E) or a phosphorylation deficient mutant (T203A) and compared to the wild-type protein (wt); recombinant GST-FBXL5 or GST were used as baits. (C) A GST-FBXL5 pull-down assay was carried out using extracts of cells co-transfected with Snail1-HA and Flag-Lats2 or control plasmid. (**D**) A nuclear export-deficient Snail1 mutant is not degraded by FBXL5. HEK293T cells were transfected with GFP-Snail1LA-HA and an empty vector or 6xMyc-FBXL5 for 24h, treated with 20 μg/ml cycloheximide for the indicated time and lysed. Snail1LA and FBXL5 levels were analysed by Western Blot. Below: that figure shows the average of the densitometric analysis of the Snail1-HA band relative to Tubulin (n=3). (**E**) *In vivo* ubiquitination was performed as in Fig. 5D using GFP-Snail1-HA and GFP-Snail1LA-HA proteins. Asterisk indicates an unspecific band.

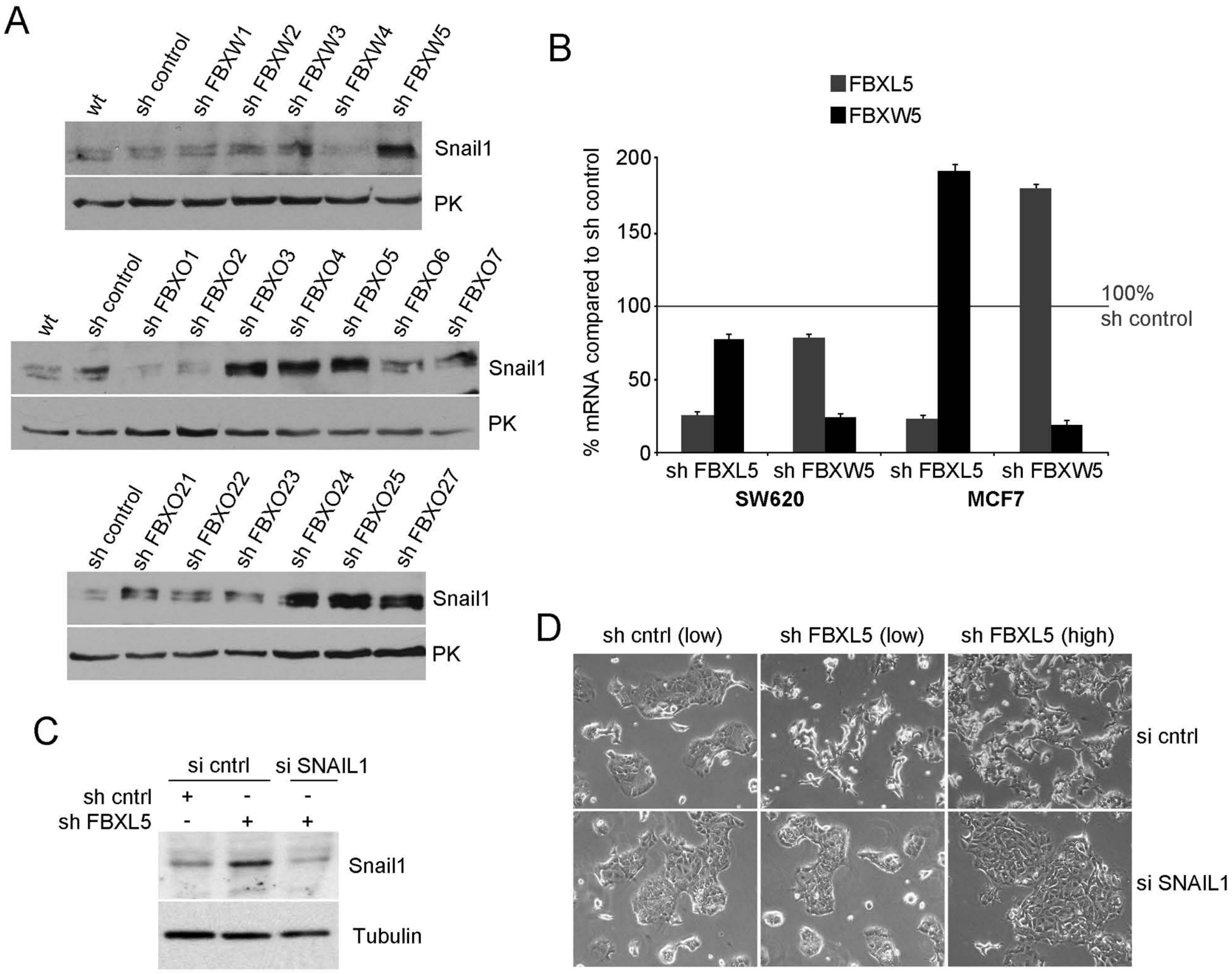

Supplementary Figure S1

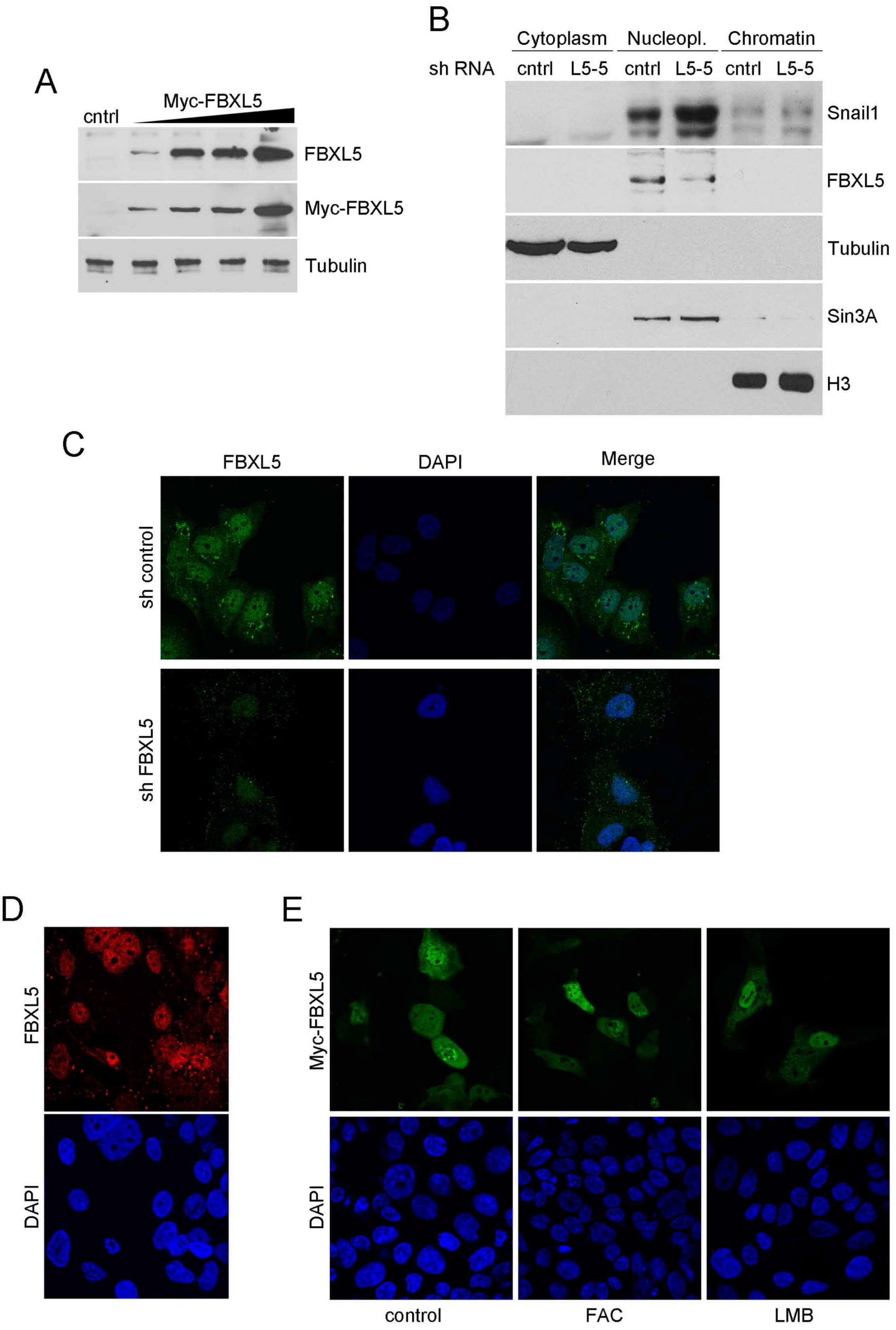

Supplementary Figure S2

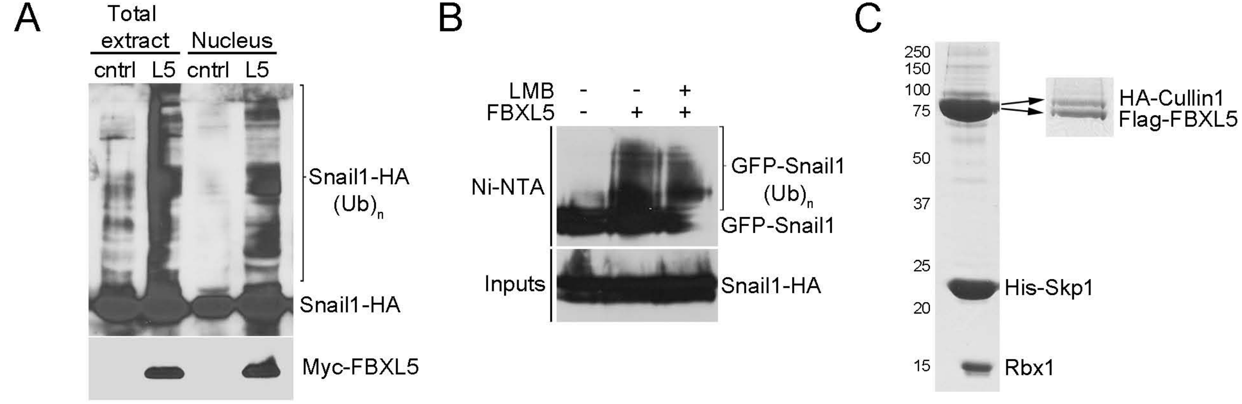

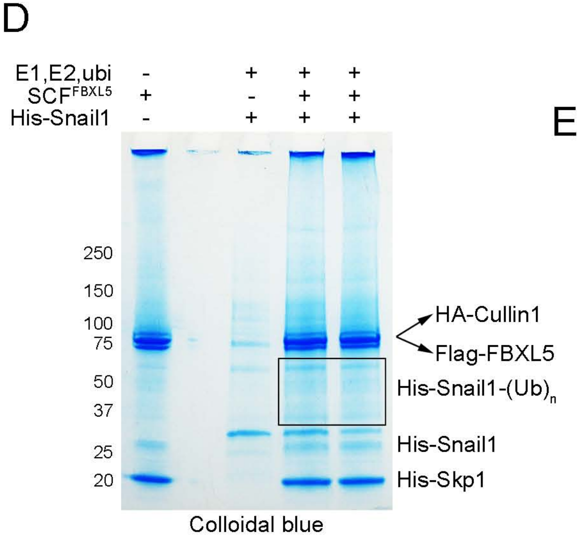

E

Peptides	Modification	aa
ESP**K**AVELTSLSDEDSGK	GlyGly(K)	K85
LSVA**K**DPQSR	GlyGly(K)	K146
AHLQTHSDV**K**R	GlyGly(K)	K234

Supplementary Figure S3

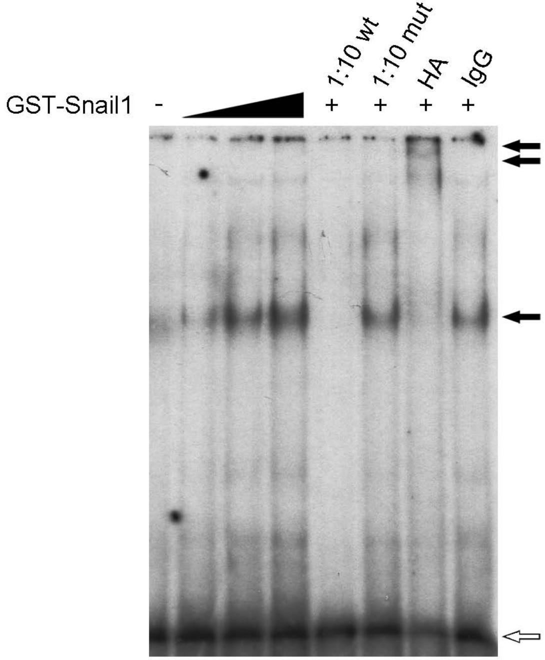

Supplementary Figure S4

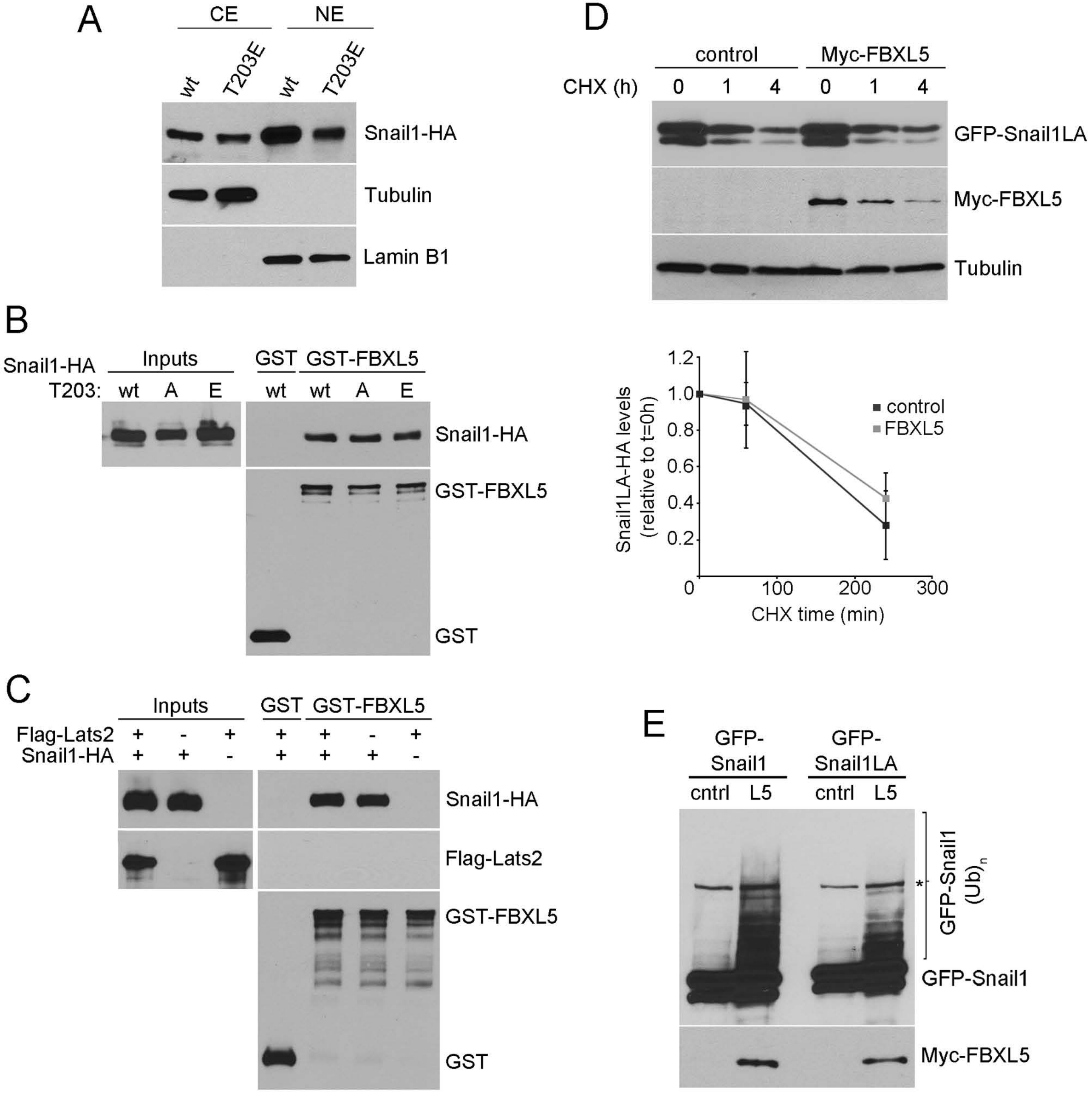

Supplementary Figure S5
