# Supplementary figures and images for: Correction to ‘Nuclear ubiquitination by FBXL5 modulates Snail1 DNA binding and stability’
Source: Nucleic Acids Res. 2026 Mar 17;54(5):gkag263. doi: 10.1093/nar/gkag263 (PMC12993436; doi:10.1093/nar/gkag263)

## Slide 1
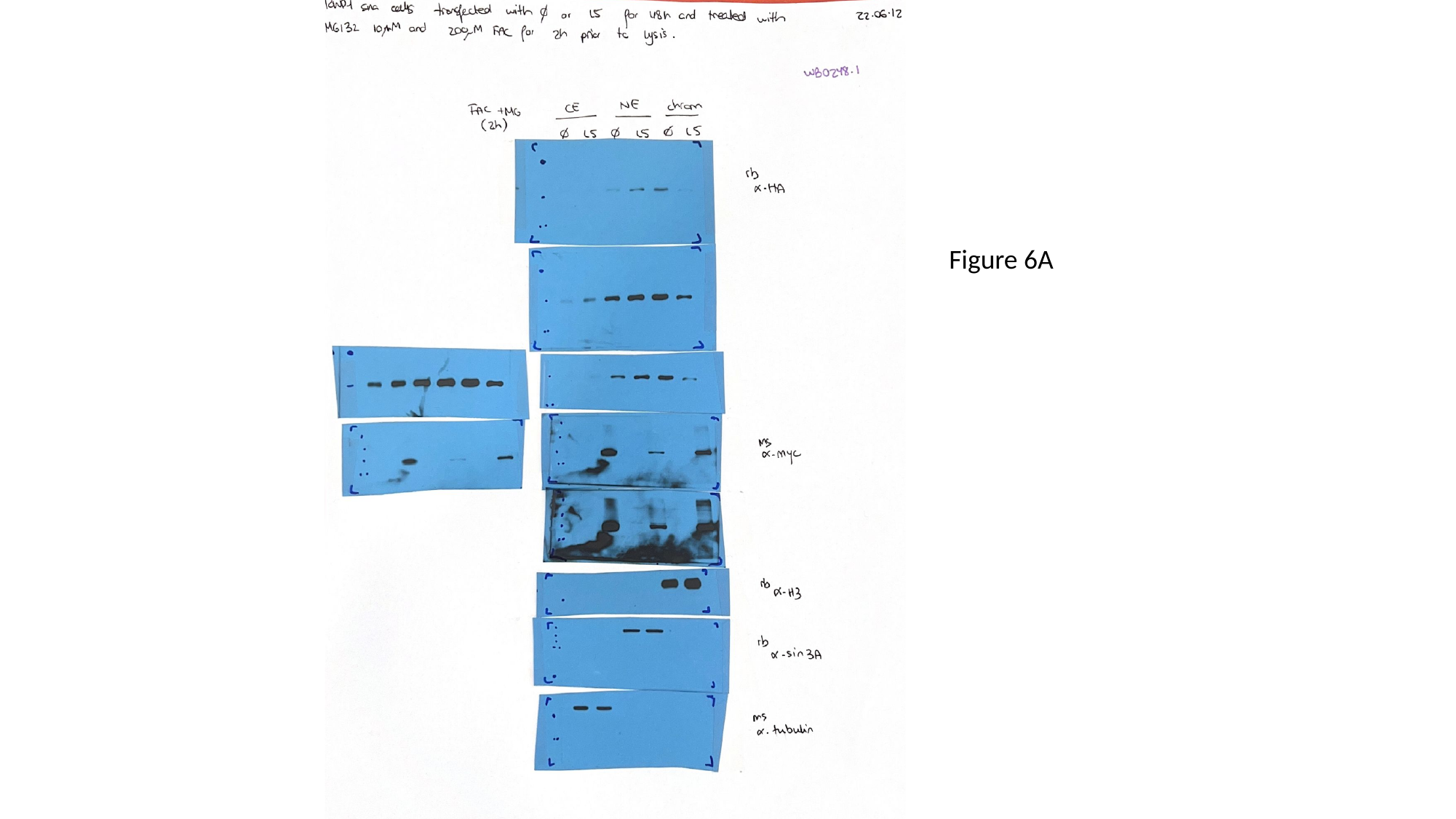

Figure 6A

## Slide 2
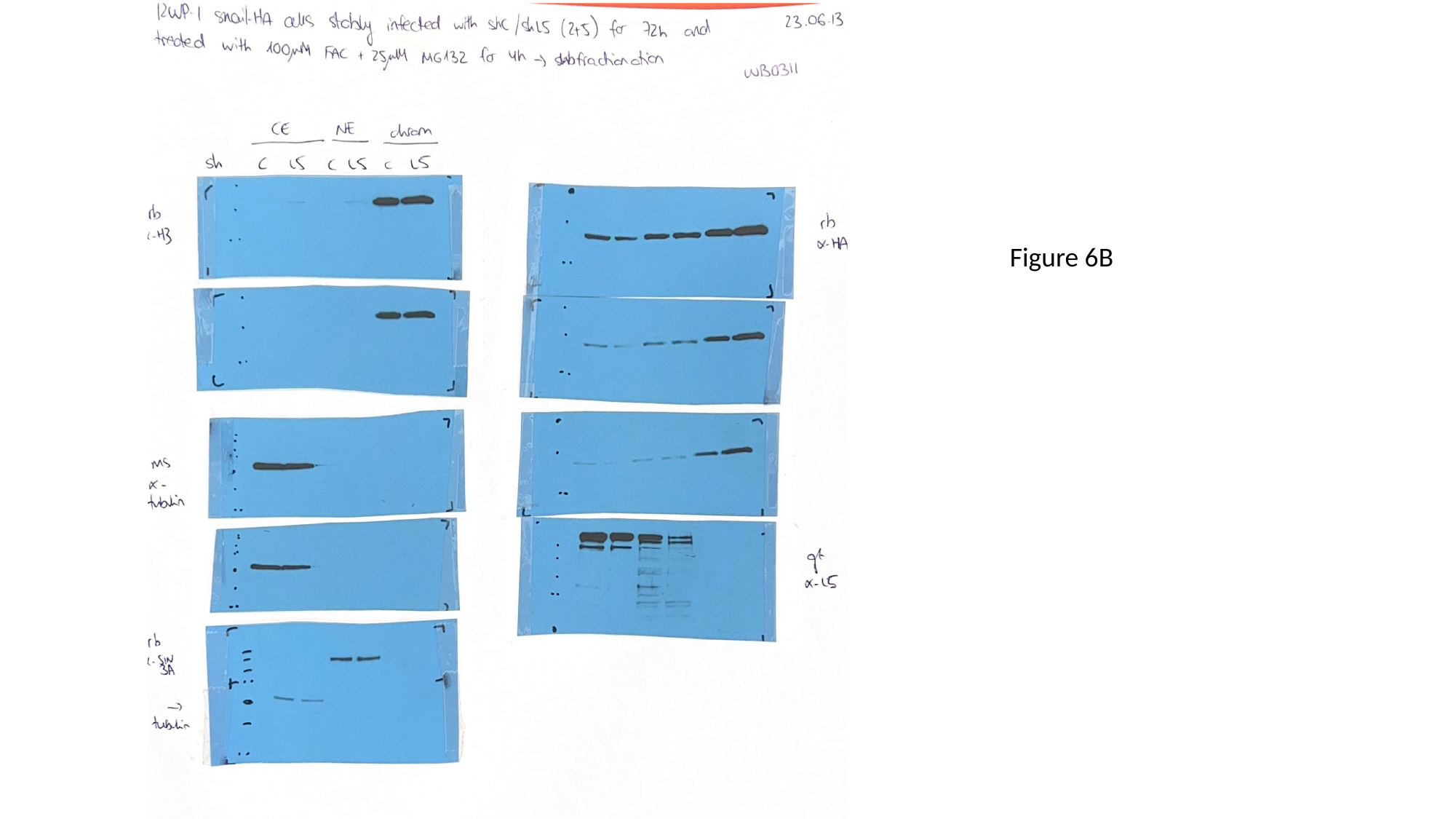

Figure 6B

## Slide 3
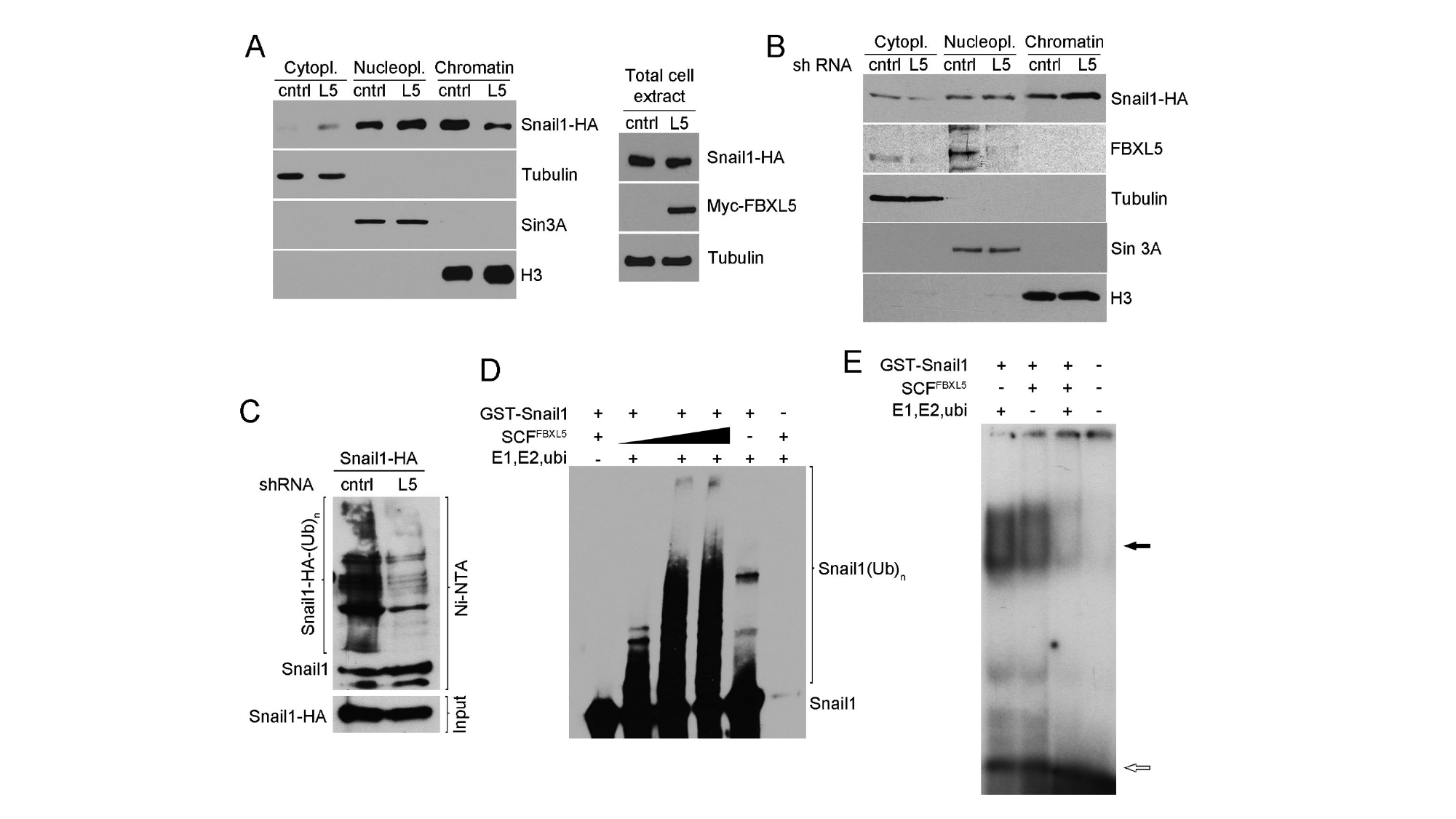

Supplement: gkag263_Supplemental_File [file gkag263_supplemental_file.pptx]
